# Reproductive isolation via polygenic local adaptation in sub-divided populations: Effect of linkage disequilibria and drift

**DOI:** 10.1371/journal.pgen.1010297

**Published:** 2022-09-01

**Authors:** Himani Sachdeva

**Affiliations:** Department of Mathematics, University of Vienna, Vienna, Austria; University of Wyoming, UNITED STATES

## Abstract

This paper considers how polygenic local adaptation and reproductive isolation between hybridizing populations is influenced by linkage disequilibria (LD) between loci, in scenarios where both gene flow and genetic drift counteract selection. It shows that the combined effects of multi-locus LD and genetic drift on allele frequencies at selected loci and on heterozygosity at neutral loci are predicted accurately by incorporating (deterministic) effective migration rates into the diffusion approximation (for selected loci) and into the structured coalescent (for neutral loci). Theoretical approximations are tested against individual-based simulations and used to investigate conditions for the maintenance of local adaptation on an island subject to one-way migration from a differently adapted mainland, and in an infinite-island population with two habitats under divergent selection. The analysis clarifies the conditions under which LD between sets of locally deleterious alleles allows these to be collectively eliminated despite drift, causing sharper and (under certain conditions) shifted migration thresholds for loss of adaptation. Local adaptation also has counter-intuitive effects on neutral (relative) divergence: *F*_*ST*_ is highest for a pair of subpopulations belonging to the same (rare) habitat, despite the lack of reproductive isolation between them.

## Author summary

Environmental adaptation often involves spatially heterogeneous selection at many genetic loci. Thus, the evolutionary consequences of hybridisation between populations adapted to different environments depend on the *coupled* dynamics of multiple loci under selection, migration and genetic drift, making them challenging to predict. Here, I introduce theoretical approximations that accurately capture the effect of such coupling on allele frequencies at individual loci, while also accounting for the stochastic effects of genetic drift. I then use these approximations to study hybridisation in a metapopulation consisting of many interconnected subpopulations, where each subpopulation belongs to one of two habitats under divergent selection. The analysis clarifies how subpopulations belonging to a rare habitat can maintain local adaptation despite high levels of migration if net selection against multi-locus genotypes is stronger than a threshold which depends on the relative abundances of the two habitats. Further, local adaptation in a metapopulation can significantly elevate *F*_*ST*_ between subpopulations belonging to the same habitat, even though these are not reproductively isolated. These findings highlight the importance of carefully considering the genetic architecture and spatial context of divergence when interpreting patterns of genomic differentiation between speciating populations.

## Introduction

Environmental adaptation in natural populations typically involves selection that varies over space and time, and acts on many genetic loci [1–4]. The ability of populations to adapt to their local environment thus depends on the ease with which locally favoured alleles can establish and be maintained across multiple loci, despite maladaptive gene flow from other regions. A key question is: to what extent does selection act on *combinations* of selected alleles, as opposed to individual alleles [5, 6]? More generally, when do linkage disequilibria (LD), i.e., statistical associations between sets of locally adaptive alleles—that may be either tightly clustered or widely distributed across the genome—protect such alleles from swamping, especially in marginal habitats, which are prone to swamping [7]?

The buildup and/or maintenance of LD between sets of divergently selected alleles may be viewed as an example of the broader process of ‘coupling’ between barrier loci, which reduces genetic exchange between populations [8, 9]—a precursor to reproductive isolation (RI) and speciation. However, many questions remain as to the role of such coupling during the initial vs. late stages of divergence, its role in allopatric vs. parapatric divergence, and in case of the latter, whether divergence (with gene flow) involves adaptation from standing genetic variation or new mutations [10].

Barton (1983) [5] considered the consequences of secondary contact between populations subject to divergent directional selection at multiple equal-effect loci, and showed that in large populations, where the effects of drift can be neglected, the strength of LD between a set of introgressing deleterious alleles depends on the selection density, i.e., the ratio of the total selection strength (against the entire set of alleles) to the total recombination rate (over the map length spanned by the alleles). If total selection is stronger than total recombination, and immigration sufficiently weak that deleterious alleles segregate at low frequencies, *sets* of such alleles are eliminated by selection much faster than they are broken apart by recombination. The frequency of any allele at migration-selection equilibrium is then influenced more by indirect selection due to LD with other deleterious alleles than direct selection due to its own deleterious effect. Conversely, when recombination is much faster than selection, allele frequencies evolve more or less independently across loci, primarily under direct selection.

Selection against introgressing deleterious genotypes also impedes neutral gene flow if neutral alleles are in strong LD with alleles that are divergently selected across populations. This reduction in neutral exchange can be quantified in terms of *effective* migration rates (in case of discrete populations connected via migration; see e.g., [11]) or the strength of a ‘barrier’ to gene flow (in case of spatially continuous populations subject to heterogeneous selection; e.g., [12]). Barton and Bengtsson (1986) [13] calculated effective migration rates and barrier strengths in large populations (i.e., neglecting drift) for a variety of multilocus configurations and spatial geometries. They showed that when barrier loci are linked across a linear genome, then the effective migration rate of a neutral allele at an arbitrary genomic location is strongly reduced only when selection density, i.e, net selection against introgressing deleterious alleles per unit map length, is high. With unlinked barrier loci, effective migration rates at neutral markers are reduced relative to the actual migration rate by a factor that depends (to a good approximation) only on the relative fitness of immigrants [11].

A more complex picture emerges for divergence with ongoing gene flow—a locally beneficial mutation that arises near a pre-existing barrier locus enjoys increased chances of establishment (so-called ‘divergence hitchhiking’; see e.g., [14]). However, this effect is typically restricted to a small map region (with a correspondingly small mutation target) around the barrier locus, and thus, does not markedly influence the rate of buildup of divergence between populations. If the genomic density of barrier loci is low (as in early stages of divergence), any newly arisen mutation will find itself in the vicinity of at most one such locus, allowing us to investigate the effects of divergence hitchhiking on the establishment of new mutations using relatively simple two-locus models [15–19].

As divergence builds up, any new mutation comes to be influenced by more barrier loci- until a critical divergence threshold is reached, beyond which there is a sharp decline in effective migration rates and a corresponding increase in establishment probabilities of locally adaptive variants across the *entire* genome [20, 21]. This has been hypothesised to cause a ‘tipping point’ in the course of speciation, with the buildup of RI rapidly accelerating beyond this point [22]. However, an important limitation of these studies is that they only consider divergence via the sequential establishment of new mutations (as opposed to response from standing genetic variation). Moreover, they rely largely on simulations, making it difficult to arrive at a complete picture of multilocus interactions during adaptive divergence.

A further complication arises when populations are small and drift comparable to selection per locus. Not only can drift, in conjunction with maladaptive gene flow, then impair the efficacy of selection at individual loci [23], it might also generate negative LD between sets of deleterious alleles via Hill-Robertson interference [24]: this may counteract positive LD due to migration, thus also compromising the efficacy of LD-driven or collective elimination of groups of alleles.

Understanding the combined effects of LD and genetic drift on local adaptation is important as extended populations are often patchworks of smaller, interconnected subpopulations. If density regulation occurs primarily within subpopulations, then the rate of drift is governed by local sizes rather than the size of the population as a whole. Additionally, if locally adaptive traits are polygenic, then individual loci contributing to trait variation may have rather weak selective effects, such that typical values of *Ns* are small, resulting in local adaptation via many small (and possibly transient) allele frequency shifts [25, 26].

This paper considers some of these issues by analysing polygenic local adaptation in a metapopulation comprised of many small subpopulations occupying different habitats, assuming that fitness is influenced by many loci with habitat-dependent selective effects. It explores conditions for local adaptation in two scenarios—first, for an island subject to maladaptive gene flow from a large and perfectly adapted mainland, and second, in an infinite-island population with two habitats subject to divergent selection. The focus is on understanding when LD between locally adaptive alleles allows adaptation to be maintained in a *rare* habitat (which encompasses a small fraction of all islands) despite migration, and to what extent the effects of LD may be washed out by drift. A key question is: How does the genetic architecture of local adaptation (i.e., the number and effect sizes of locally adaptive variants) influence evolutionary outcomes in a scenario where adaptation involves response from high levels of standing genetic variation? I further explore how neutral diversity in either habitat is influenced by the extent of adaptive divergence, and how this translates into expectations for various *F*_*ST*_ measures.

The paper also illustrates how the gross effects of multi-locus LD and genetic drift at any individual selected locus are accurately predicted by incorporating appropriately defined effective migration rates (for selected alleles) into the single-locus diffusion approximation (see e.g., [27]). Analogously, the effects of LD on neutral diversity within any subpopulation are captured by incorporating effective migration rates (for neutral alleles) into the structured coalescent. While the basic approach of splicing effective migration rates into the single-locus diffusion approximation has been employed in earlier work on two-locus models [28–32], as we see below, this approach turns out to be especially powerful when many loci (spread across the entire genome) are involved in divergence: the effective migration rate at any locus is then roughly independent of its own effect, depending instead on the relative fitness of migrant individuals, which can often be estimated in the field, e.g., in reciprocal transplant experiments [33], or from pedigrees [34].

More generally, a mathematical understanding of multi-locus evolution under selection and drift remains elusive, despite the centrality of such an understanding to fundamental evolutionary questions regarding the limits to natural selection, the evolution of sex and recombination, and the maintenance of genetic variation. Thus, heuristic approximations (of the kind developed here) can play an important role in our understanding of stochastic effects during polygenic adaptation [33, 35–37].

## Models and methods

### Mainland-island model

Consider an island with *N* haploid individuals, subject to ongoing migration from a large mainland. Island and mainland populations are under divergent selection at *L* unlinked, biallelic loci. Selection is multiplicative across loci, with different alleles favoured on the mainland and island at each locus, independent of the state of other loci. For simplicity, we will also take effect sizes to be equal across loci. Then, the fitness of any individual on the island depends only on *y*, the number of locally deleterious alleles it carries, and *s*, the selective effect per deleterious allele, and is given by *W*(*y*)=*e*^−*sy*^.

We assume that the mainland population is fixed at all selected loci for the allele that is deleterious on the island, so that immigrants have fitness proportional to *e*^−*sL*^. Polymorphism can still be maintained on the island despite drift and continual gene flow from the mainland, provided mutation rates are above some threshold value.

The lifecycle on the island is as follows: in each generation, a Poisson-distributed number of individuals (on average *Nm*) are replaced by migrants from the mainland, where *m* is the migration rate. Individuals then undergo mutation, with rate of mutation *μ* between alternative alleles per locus per individual. Following mutation, the next generation is formed by sampling 2*N* parents (with replacement) with probabilities proportional to their relative fitness. Finally, the 2*N* parents are paired randomly and recombinant haploid offspring created via free recombination between pairs.

### Infinite-island model

Consider a population with *D* islands, where each island has *N* haploid individuals. In theoretical analyses, we will assume *D* → ∞, i.e., consider the infinite-island model [27]. The fitness of an individual depends on the local environment or *habitat* on the island on which it resides. For simplicity, we will assume only two habitats, with a fraction *ρ* of islands supporting the first habitat, and the remaining fraction 1−*ρ* the second. Assuming *ρ*<1/2, the first habitat is always ‘rare’ (i.e., it characterizes a minority of islands in the population) and the second ‘common’.

As before, fitness is influenced by *L* unlinked, biallelic, equal-effect loci, with alternative alleles favoured in the two habitats at each locus. The relative fitness of an individual carrying *i* *locally* deleterious alleles in a deme belonging to habitat *k* is $e^{-s_{k}i}$, where *s*_*k*_ is the selective effect per locally deleterious allele in habitat *k*. We will use the subscripts *r* and *c* to denote the rare and common habitats. For simplicity, we will only consider symmetric selection (with *s*_*r*_=*s*_*c*_=*s*), but the approximations described below apply more generally.

With an infinite number of demes, the mutation rate can be set to zero, since mutation is not essential for the maintenance of polymorphism as long as *ρ* is not vanishingly small, and because we are primarily interested in the effect of gene flow on local adaptation from standing variation. The extension to non-zero mutation rates is straightforward.

In each generation and in each deme, a Poisson-distributed number of individuals (with mean *Nm*) are replaced by migrants from a common pool, which is formed by drawing individuals uniformly from across all demes. Following migration, the next generation is formed by randomly sampling 2*N* parents (within each deme) with probabilities proportional to local relative fitness. *N* haploid offspring are then created in each deme by free recombination between parental pairs. For the infinite-island model, we will also follow neutral markers that are unlinked to any selected locus and to each other—this allows us to investigate how the extent of adaptive divergence between habitats influences neutral gene flow and genome-wide RI.

Since the main goal is to clarify how LD and drift jointly influence local adaptation in a structured population, other kinds of complexity are neglected. Organisms are assumed to be haploid (thus neglecting the effects of dominance). Loci are assumed to be unlinked and selective effects taken to be the same for all loci. More crucially, the model assumes an extreme form of divergent selection, wherein any selected allele has opposite effects in the two habitats (regardless of alleles at other loci). Finally, there is no explicit space and no isolation-by-distance.

In the following, I briefly outline theoretical approximations that predict allele frequency divergence in different limiting cases: I first discuss the single-locus diffusion approximation for allele frequencies in a subdivided population [27]– this accounts for the effects of genetic drift but neglects LD. I then discuss deterministic analyses that account for LD but neglect drift [5, 13]; it is useful to represent the effects of LD in such analyses by an effective migration rate for the selected or neutral allele [11, 28]. Finally, I describe how effective migration rates can be incorporated into the diffusion approximation, leading to novel approximations that accurately predict allele frequency divergence in parameter regimes where both multi-locus LD and drift play a role.

### Diffusion approximation (assuming LE)

If net selection against maladapted genotypes is weak relative to recombination, then LD between selected variants can be neglected and loci assumed to evolve independently, i.e., under linkage equilibrium (LE). For definiteness, we will use *p* to denote the frequency of the allele that is locally disadvantageous in the rare habitat (or in case of mainland-island migration, on the island) and thus advantageous in the common habitat (or on the mainland, where we assume *p*=1). If 1/*N*, *s*, *m*, *μ*≪1, then the probability distribution *ψ*[*p*] of the allele frequency *p* at any locus under mutation-selection-migration-drift equilibrium is predicted by the diffusion approximation, and depends only on the scaled parameters *Ns*, *Nμ* and *Nm* [27].

The equilibrium frequency distribution on an island subject to one-way migration from the mainland is given by (see e.g., [27] for details):

$$\begin{array}{c} \psi_{island}\left[p\right]\;\;\propto \;\;p^{2Nm+2N\mu -1}{\left(1-p\right)}^{2N\mu -1}e^{-2Nsp} \end{array}$$ (1)

Integrating over the normalised distribution gives the expected allele frequency E[p].

For the infinite-island model, one can express the allele frequency distribution $\psi_{i}\left[p|\bar{p}\right]$ at any locus in a deme within habitat *i*, as a function of $\bar{p}$, the allele frequency at that locus in the migrant pool [27]:

$$\begin{array}{c} \psi_{i}\left[p|\bar{p}\right]\;\;\propto \;\;p^{2Nm\bar{p}-1}{\left(1-p\right)}^{2Nm(1-\bar{p})-1}{\bar{W}}_{i}^{2N} \end{array}$$ (2)

where ${\bar{W}}_{i}$ is the mean population fitness for a deme in habitat *i*. The subscript *i* can take on values *r* and *c*, corresponding to the rare and common habitats respectively. We have: ${\bar{W}}_{r}\;=\;e^{-s\;p}$ and ${\bar{W}}_{c}\;=\;e^{-s\;(1-p)}$, since alternative alleles are favoured in the two habitats. One can now calculate the expected allele frequency $E[p_{i}|\bar{p}]$ in either habitat as a function of $\bar{p}$ by integrating over the frequency distribution above. At equilibrium, $\bar{p}$ must be equal to the expected allele frequency across the entire population. This allows us to obtain $\bar{p}$ by numerically solving $\bar{p}=\left(1-\rho \right)E\left[p_{c}|\bar{p}\right]+\rho E\left[p_{r}|\bar{p}\right]$ (see also [38]).

### Effective migration rates (neglecting drift)

The assumption of LE is valid only if recombination is faster than all other evolutionary processes. In particular, this requires *Ls*≪1/2, i.e., the net selective disadvantage of maladapted immigrant genotypes must be much weaker than recombination. Conversely, with strong selection against immigrants i.e., *Ls*≳1/2, sets of incoming alleles are eliminated together before recombination can split them, causing allele frequencies across different loci to evolve in a coupled manner. Thus, in this regime, we must explicitly consider multilocus dynamics in order to account for the effects of LD on allele frequencies [5].

Consider a large population that receives migrants, carrying *L* unlinked deleterious alleles, at a steady rate *m* per generation. Let {*P*_*y*_} denote the frequencies of genotypes with *y*=1, 2, …*L* deleterious alleles in the population at equilibrium. If selection per locus and migration are much stronger than drift, i.e., 1/*N*≪*m*∼*s*≪*Ls*∼1, then genotype frequencies evolve essentially deterministically. Further, if deleterious genotypes are rare at equilibrium (e.g., as expected for large *Ls*), then mating between individuals both carrying deleterious genotypes can be neglected. Under these conditions, {*P*_*y*_} satisfy the following coupled *linear* equations:

$$\begin{array}{c} P_{y}=[m\;w_{L}\left(\begin{array}{c} L\\ y \end{array}\right){\left(\frac{1}{2}\right)}^{L}+\sum_{k=y}^{L}P_{k}w_{k}\left(\begin{array}{c} k\\ y \end{array}\right){\left(\frac{1}{2}\right)}^{k}]\;\text{where}\;w_{i}=2e^{-is} \end{array}$$ (3)

Here, *w*_*k*_=2*e*^−*ks*^ is the average number of offspring of an individual carrying *k* deleterious alleles, and $\left(\begin{array}{c} k\\ y \end{array}\right){\left(\frac{1}{2}\right)}^{k}$ the probability that the individual transmits exactly *y* of these deleterious alleles to an offspring. Note that for *s*=0, individuals have an average of 2 offspring and transmit half of their genome to any offspring on average.

Eq (3) can be solved to obtain the average deterministic deleterious allele frequency $p_{det}=\left(\sum_{y=1}^{L}y\;P_{y}\right)/L\;$ (Section 1 in S1 Text). One can then use this to define the effective migration rate *m*_*e*_[*s*, *L*] for a selected allele as that rate of migration which would cause the allele frequency at a *single* locus under migration-selection balance (with selective disadvantage *s* for the deleterious allele) to be the same as *p*_*det*_, the average deleterious frequency that emerges in the multi-locus model, where immigrant genotypes carrying *L* such deleterious alleles are introduced at rate *m* per generation. Then, we have (see Section 1 in S1 Text):

$$\begin{array}{c} \frac{m_{e}\left[s,L\right]}{m}={\left(\frac{e^{-s}}{2-e^{-s}}\right)}^{L-1}\left(1-e^{-s}\right)\sum_{k=0}^{\infty }e^{-s\;k}{\left(1+\frac{e^{-sk}}{2^{k}}-\frac{e^{-s\left(k+1\right)}}{2^{k}}\right)}^{L-1} \end{array}$$ (4)

Following [13], we can also calculate an effective migration rate $m_{e}^{*}\left[s,L\right]$ for neutral alleles. By definition, $m_{e}^{*}\left[s,L\right]/m$ is the probability that the neutral allele escapes via one or more recombination events from the immigrant genetic background (which has an excess of *L* deleterious alleles of effect *s*) onto a resident background, before it is lost from the population. For an unlinked neutral allele, this is (Section 1 in S1 Text):

$$\begin{array}{c} \frac{m_{e}^{*}\left[s,L\right]}{m}={\left(\frac{e^{-s}}{2-e^{-s}}\right)}^{L} \end{array}$$ (5)

In the limit *s*→0, *L*→∞, with *Ls* constant, i.e., assuming that a given total selective disadvantage *Ls* is due to larger and larger numbers of loci of weaker effects, the two (scaled) effective migration rates, *m*_*e*_[*s*, *L*]/*m* and $m_{e}^{*}\left[s,L\right]/m$, can be approximated as (see Section 1 in S1 Text):

$$\begin{array}{ccc} \frac{m_{e}\left[s,L\right]}{m}\; & =\;e^{-2\theta }[1+s\left(\theta +\sum_{k=0}^{\infty }\left(e^{\frac{\theta }{2^{k}}}-1\right)\right)+O\left(s^{2}\right)]\;\\ & \approx \;e^{-2\theta }[1+s\left(e^{\theta }+e^{\theta /2}+\frac{3}{2}\theta -2\right)+O\left(s^{2}\right)]\;\;\theta \;=\;\left(L-1\right)s & \text{(6a)}\\ & \frac{m_{e}^{*}\left[s,L\right]}{m}\;\approx \;e^{-2\theta_{*}}[1+s\;\theta_{*}+O\left(s^{2}\right)+\ldots ]\;\;\theta_{*}\;=\;Ls & \text{(6b)} \end{array}$$

Note that in this limit, we also have *θ*→θ_*_. However, I still distinguish between the two to highlight the conceptual distinction that the barrier effect at any selected locus is due to the other *L*−1 selected loci, while the barrier effect at a neutral locus is due to *L* selected loci. Fig A in S1 Text illustrates how the ratios *m*_*e*_[*s*, *L*]/*m* and $m_{e}^{*}\left[s,L\right]/m$ converge towards the large-*L*/small-*s* predictions of Eq (6) for various values of *Ls*.

We can see from Eq (6) (see also Fig A in S1 Text) that *m*_*e*_/*m* is lowest or the barrier effect *strongest* when the same total selective disadvantage *Ls* is due to a very large number of loci of very weak effect. In this limit, the effective migration rate of any allele (neutral or selected) is reduced relative to *m* by approximately *e*^−2*θ*^, where *θ* is the net selective disadvantage (in the recipient population) of the genetic background of the immigrating allele. One can also arrive at this result in a less rigorous but more general way from the fact that $m_{e}^{*}/m$ for a neutral unlinked allele (in the limit of weak migration) is equal to the average *reproductive value* (RV) of migrants [39]. Here, RV refers to the migrant’s long-term genetic contribution to the recipient population [40]. An approximate expression for the RV of the migrant in the highly polygenic limit can be derived as follows (see also [11]).

Let *K* denote the average number of locally deleterious alleles per genome in the resident population. Migrants carry an excess of *L*−*K* deleterious alleles with respect to the average resident, and their relative fitness is *e*^−(*L*−*K*)*s*^ (neglecting fitness variance within the resident population). Since the immediate progeny of the migrant (i.e., *F*_1_ individuals) will carry $\frac{L+K}{2}$ deleterious alleles on average, i.e., an excess of $\frac{L-K}{2}$ deleterious alleles, their relative fitness is ≈*e*^−*s*(*L*−*K*)/2^. For *m*≪1 (i.e., when mating between individuals with recent immigrant ancestry can be neglected), most second-generation descendants of the immigrant individual are first-generation backcrosses; thus, they carry an excess of $\frac{L-K}{4}$ deleterious alleles on average and have relative fitness ≈*e*^−*s*(*L*−*K*)/4^. Similarly, third-generation descendants would have fitness ≈*e*^−*s*(*L*−*K*)/8^, and so on. Thus, the average RV of a migrant, which is the product of its own relative fitness with that of all its descendants, is approximately *e*^−*s*(*L*−*K*)^ *e*^−*s*(*L*−*K*)/2^ *e*^−*s*(*L*−*K*)/4^ *e*^−*s*(*L*−*K*)/8^…=*e*^−2*s*(*L*−*K*)^. Conceptually similar arguments (based on tracking the long-term genetic contribution of individuals in a population) have also been used to derive how fitness variance at unlinked loci affects effective population size [41].

Note that we recover *m*_*e*_/*m* ≈ *e*^−2*Ls*^ (as in Eq (6) above) for very rare migration, i.e., when most genotypes in the resident population have no deleterious alleles (*K*≈0). This derivation is only approximate as it neglects the segregation variance among descendants of the migrant individual as well as fitness variance in the resident population. The derivations based on Eq (3) (see also eq. 6 in S1 Text) account for the former by summing over the frequencies of all possible offspring genotypes. However, this only leads to corrections that are O(s) and are thus small for weak per-locus selection.

### Introducing effective migration rates into the diffusion approximation

If individual subpopulations are small, drift may be comparable to selection per deleterious allele, but much weaker than selection against immigrant genotypes (that carry many such alleles). This corresponds to a parameter regime with 1/*N*∼*m*∼*s*≪*Ls*∼1. In this case, genotypes with recent immigrant ancestry, that carry a *large* excess (i.e., O(L)) of deleterious alleles relative to the average resident, exhibit essentially deterministic evolutionary dynamics. By contrast, more ‘average’ genotypes (that carry a *small* excess or deficit of deleterious alleles relative to the mean) will have dynamics that are significantly perturbed by drift.

Thus we have the following heuristic picture: a small subset of deleterious alleles embedded in immigrant or early-backcross genotypes experience strong negative selection due to statistical associations with other such alleles, but are largely unaffected by drift. Since such genotypes are rapidly broken down by recombination (at least under free recombination) and/or eliminated by strong selection, they contribute little to fitness variance in the recipient population. As a consequence, the *majority* of deleterious alleles are embedded in more average genotypes and are, thus, significantly affected by direct selection (due to their own deleterious effect) and genetic drift, but not indirect selection due to LD. Under these conditions, it is reasonable to expect that the equilibrium allele frequency distribution would be close to the single-locus distribution under drift-migration-selection-mutation balance, but with a *reduced* effective rate of migration, where the reduction reflects selection against immigrant and early-backcross genotypes, which causes sets of deleterious alleles to be eliminated together.

To make these arguments more concrete, consider first the mainland-island case. If the rate of drift 1/*N* is much higher than the (effective) rate of migration and mutation, allele frequency distributions will be U-shaped, and the island population near fixation for one or other allele at each locus. Denoting the expected deleterious allele frequency per locus under migration-selection-mutation-drift balance by E[p], a U-shaped distribution implies that the island is near fixation for the locally deleterious allele at ≈LE[p] loci, so that migrants from the mainland carry an excess of L(1-E[p]) alleles on average. This suggests that the relevant reduced migration rate governing allele frequency distributions (and consequently the expected frequency E[p]) can be approximated by $m_{e}\left[\;s,L\left(1-E\left[p\right]\right)\;\right]$, where *m*_*e*_ is given by Eq (4).

We use $\psi_{island}\;[\;p\;|\;E[p]\;]$ to denote the allele frequency distribution, conditional on the expected allele frequency E[p]. This is simply given by Eq (1), but with the raw migration rate *m* replaced by the effective migration rate $m_{e}\left[\;s,L\left(1-E\left[p\right]\right)\;\right]$. We can now obtain an implicit equation for E[p] by using the fact that: $E\left[p\right]=\int dp\;\psi_{island}\;[\;p\;|\;E[p]\;]$. Thus, we have:

$$\begin{array}{c} E\left[p\right]=2N\left(\mu +m_{e}\right)\frac{{\;}_{1}{\tilde{F}}_{1}\left(1+2Nm_{e}+2N\mu ,\;1+2Nm_{e}+4N\mu ,\;-2Ns\right)}{{\;}_{1}{\tilde{F}}_{1}\left(2Nm_{e}+2N\mu ,\;2Nm_{e}+4N\mu ,\;-2Ns\right)},\;m_{e}=m_{e}\left[\;s,L\left(1-E\left[p\right]\right)\;\right] \end{array}$$ (7)

where ${}_{1}{\tilde{F}}_{1}$ is the regularized confluent hypergeometric function of the first kind. Eq (7) can be solved numerically to obtain E[p].

Note that in replacing *m* (which is the migration rate *per generation*) by *m*_*e*_ (which is a composite parameter that encapsulates the splitting of the migrant genome over multiple, i.e., ∼10 generations of backcrossing), we implicitly assume that the splitting occurs much faster than changes due to any single-locus process, i.e., 1/*s*, 1/*m*, *N* ≪ 1.

When allele frequency distributions at selected loci are not U-shaped and heterozygosity E[pq] is appreciable, then the average fitness on the island is $e^{-LsE\left[p\right]+L\frac{s^{2}}{2}E\left[pq\right]}$. Thus, the relative selective disadvantage (and RV) of a migrant on the island will depend not only on the average number of selective differences between the mainland and island populations, but also on the heterozygosity (at selected loci) within the island population. However, the term involving heterozygosity is proportional to *s*^2^ *L* and is thus much smaller (by a factor that is O(s)) than the first term involving the expected allele frequency, provided individual selective effects are weak. Thus, we can neglect it to a first approximation.

Now consider the case of the infinite-island population with two habitats. If both habitats are locally adapted, then individuals migrating between demes within the same habitat will have higher RV, i.e., contribute more genetic material to future generations, than individuals migrating between demes belonging to different habitats. Thus, deleterious alleles can be associated with effective migration rates that now depend on both the habitat from which the allele originates as well as the habitat into which it immigrates.

Let ${\bar{p}}_{r}$ and ${\bar{p}}_{c}$ denote the expected frequencies in the rare and common habitat respectively (for the allele that is favoured in the common habitat). If drift is strong or at least comparable to other evolutionary processes, then any deme is close to fixation for one or other allele at each locus. In this scenario, immigrants will typically carry alleles that are positively selected vis-a-vis the resident allele at some loci and negatively selected at other loci. In principle, one can calculate the deterministic introgression dynamics of such a mosaic genome, containing both types of alleles [42]. However, as a first approximation, we will assume that all that matters is the net selective disadvantage of such a genome, which is, on average, proportional to the excess number of locally deleterious alleles that it carries, relative to a typical resident. This is $∼sL\left({\bar{p}}_{c}-{\bar{p}}_{r}\right)$ for individuals migrating *between* habitats, and zero for migrants *within* the same habitat.

As before, we can write down the distribution $\psi_{i}\left[\;p\;|\;{\bar{p}}_{r},{\bar{p}}_{c}\;\right]$ of allele frequencies on any island within habitat *i*, conditional on ${\bar{p}}_{r}$ and ${\bar{p}}_{c}$, the average frequencies across the rare and common habitats respectively. Integrating over these yields $E_{i}\left[p\;|\;{\bar{p}}_{r},{\bar{p}}_{c}\right]$, the expected frequency within any deme in habitat *i*, conditional on ${\bar{p}}_{r}$ and ${\bar{p}}_{c}$. Finally, by using the fact that ${\bar{p}}_{i}=E_{i}\left[p|{\bar{p}}_{r},\;{\bar{p}}_{c}\right]$ for all *i* (at equilibrium), we arrive at the following coupled equations for ${\bar{p}}_{r}$ and ${\bar{p}}_{c}$:

$$\begin{array}{ccc} {\bar{p}}_{r} & =E_{r}\left[p|{\bar{p}}_{r},{\bar{p}}_{c}\right]\\ & =2N\left(m_{e}^{\left(rr\right)}{\bar{p}}_{r}+m_{e}^{\left(cr\right)}{\bar{p}}_{c}\right)\\ & \;\frac{{}_{1}{\tilde{F}}_{1}[1+2N\left(m_{e}^{\left(rr\right)}{\bar{p}}_{r}+m_{e}^{\left(cr\right)}{\bar{p}}_{c}\right),1+2N\left(m_{e}^{\left(rr\right)}+m_{e}^{\left(cr\right)}\right),2Ns]}{{}_{1}{\tilde{F}}_{1}[2N\left(m_{e}^{\left(rr\right)}{\bar{p}}_{r}+m_{e}^{\left(cr\right)}{\bar{p}}_{c}\right),2N\left(m_{e}^{\left(rr\right)}+m_{e}^{\left(cr\right)}\right),2Ns]} & \text{(8a)}\\ {\bar{p}}_{c} & =E_{c}\left[p|{\bar{p}}_{r},{\bar{p}}_{c}\right]\\ & =2N\left(m_{e}^{\left(rc\right)}{\bar{p}}_{r}+m_{e}^{\left(cc\right)}{\bar{p}}_{c}\right)\\ & \;\frac{{}_{1}{\tilde{F}}_{1}[1+2N\left(m_{e}^{\left(rc\right)}{\bar{p}}_{r}+m_{e}^{\left(cc\right)}{\bar{p}}_{c}\right),1+2N\left(m_{e}^{\left(rc\right)}+m_{e}^{\left(cc\right)}\right),-2Ns]}{{}_{1}{\tilde{F}}_{1}[2N\left(m_{e}^{\left(rc\right)}{\bar{p}}_{r}+m_{e}^{\left(cc\right)}{\bar{p}}_{c}\right),2N\left(m_{e}^{\left(rc\right)}+m_{e}^{\left(cc\right)}\right),-2Ns]} & \text{(8b)}\\ & m_{e}^{\left(rr\right)}=\rho m\;m_{e}^{\left(cc\right)}=\left(1-\rho \right)m\\ & m_{e}^{\left(rc\right)}=\rho m_{e}\left[s,L\left({\bar{p}}_{c}-{\bar{p}}_{r}\right)\right]\;m_{e}^{\left(cr\right)}=\left(1-\rho \right)m_{e}\left[s,L\left({\bar{p}}_{c}-{\bar{p}}_{r}\right)\right] & \text{(8c)} \end{array}$$

Thus, in this case, allele frequency distributions depend on an effective migration *matrix* with elements $m_{e}^{\left(ij\right)}$, which denote the probabilities that a lineage sampled in habitat *j* in the present has originated from habitat *i* in the previous time step. For *i*=*j*, i.e., migration within the same habitat, $m_{e}^{\left(ij\right)}$ is assumed to be equal to the raw migration rate multiplied by the fraction of islands in the habitat of origin. For *i*≠*j*, i.e., migration between habitats, $m_{e}^{\left(ij\right)}$ is approximated by the effective migration rate $m_{e}\left[s,L\left({\bar{p}}_{c}-{\bar{p}}_{r}\right)\right]$ times the fraction of islands in the habitat of origin (see Eq (8c)). Eq (8) can be solved numerically to obtain ${\bar{p}}_{r}$ and ${\bar{p}}_{c}$—the expected frequencies in the two habitats at equilibrium.

As in the mainland-island case, we neglect the contribution of heterozygosity (within demes) to effective migration rates, as this is much smaller than the contribution of the mean allele frequency difference ${\bar{p}}_{c}-{\bar{p}}_{r}$ between demes. By the same token, in this first approximation, we can also neglect the contribution of the variance of the allele frequency difference (across different loci). As before, the contribution of these variances is smaller (by a factor that is O(s)) than the contribution of the mean allele frequency difference.

### Barriers to gene flow and neutral divergence in the infinite-island model

Local adaptation elevates genomewide *F*_*ST*_ across all subpopulations by reducing the *effective* rate of immigration into any deme (since immigrants originating from the dissimilar habitat have low RV). However, effective immigration is more strongly reduced for demes in the rare than in the common habitat, since most immigrants into the former originate from a dissimilar habitat. Thus, we must consider habitat-specific statistics $F_{ST}^{\left(r\right)}$ and $F_{ST}^{\left(c\right)}$—these represent the probability of identity by descent (at an unlinked neutral locus) of two lineages sampled from a deme within the rare and common habitats respectively, relative to the probability of identity of two lineages sampled from anywhere within the entire population.

Following Slatkin (1991) [43], we can express the expected value of these F-statistics in terms of expected pairwise coalescence times: $F_{ST}^{\left(r\right)}=1-T_{r}/T_{tot}$ and $F_{ST}^{\left(c\right)}=1-T_{c}/T_{tot}$, where *T*_*r*_, *T*_*c*_ and *T*_*tot*_ denote the average coalescence time of two lineages, both sampled from a single deme belonging to the rare habitat (*T*_*r*_), or both from a single deme within the common habitat (*T*_*c*_), or each lineage sampled independently from across the entire population (*T*_*tot*_).

Often, in practice, it is only possible to estimate neutral divergence between *pairs* of subpopulations, here labeled *i* and *j*. In this case, the expected $F_{ST}^{\left(i,j\right)}$ is: $1-\frac{\left(T_{ii}+T_{jj}\right)/2}{\left(T_{ii}+T_{jj}+2T_{ij}\right)/4}$, where *T*_*ii*_, *T*_*jj*_ and *T*_*ij*_ are, respectively, the expected coalescence times for pairs of lineages both sampled from deme *i*, or from deme *j*, or one from *i* and one from *j*. As before, we must separately consider the cases where both sampled demes belong to the rare habitat or to the common habitat or one to the rare and the other to the common habitat: this leads to three divergence measures $F_{ST}^{\left(rr\right)}$, $F_{ST}^{\left(cc\right)}$ and $F_{ST}^{\left(rc\right)}$

Let *m*_*rc*_ and *m*_*rr*_ denote the probability per unit time that a lineage sampled in the rare habitat traces back to the rare and common habitat respectively. We can define analogous backward migration rates *m*_*cc*_ and *m*_*cr*_ for lineages sampled in the common habitat. We can then obtain the various expected coalescence times using the structured coalescent (Section 2 in S1 Text), and express these in terms of the population-size-scaled migration rates *M*_*rr*_=*Nm*_*rr*_, *M*_*rc*_=*Nm*_*rc*_, *M*_*cc*_=*Nm*_*cc*_, *M*_*cr*_=*Nm*_*cr*_. As before, the key approximation is to assume that *m*_*rc*_ and *m*_*cr*_ are attenuated by a factor that depends on the ‘barrier effect’ of the *L* selected loci, when the habitats are (partially) locally adapted (see also [19] for a similar approximation). More specifically, we will assume that *M*_*rc*_ and *M*_*cr*_ are proportional to $m_{e}^{*}\left[s,L\left({\bar{p}}_{c}-{\bar{p}}_{r}\right)\right]$, where $m_{e}^{*}$ is given by Eq (5), and ${\bar{p}}_{r}$ and ${\bar{p}}_{c}$ are obtained from Eq (8). Thus, we have (see Section 2 in S1 Text for details):

$$\begin{array}{cc} F_{ST}^{\left(r\right)}=\frac{1}{1+2M_{rc}+2M_{rr}} & \text{(9a)}\\ F_{ST}^{\left(c\right)}=\frac{1}{1+2M_{cr}+2M_{cc}} & \text{(9b)}\\ F_{ST}^{\left(rr\right)}=\frac{1}{1+4M_{rc}+4M_{rr}} & \text{(9c)}\\ F_{ST}^{\left(rc\right)}=\frac{1+\left(M_{rc}+M_{rr}\right)+\left(M_{cr}+M_{cc}\right)}{1+3\left(M_{rc}+M_{rr}\right)+3\left(M_{cr}+M_{cc}\right)+8\left(M_{rc}+M_{rr}\right)\left(M_{cr}+M_{cc}\right)} & \text{(9d)}\\ F_{ST}^{\left(cc\right)}=\frac{1}{1+4M_{cr}+4M_{cc}} & \text{(9e)}\\ \begin{array}{cc} M_{rr}=\rho Nm & M_{rc}=\left(1-\rho \right)Nm_{e}^{*}[s,L\left({\bar{p}}_{c}-{\bar{p}}_{r}\right)]\\ M_{cr}=\rho Nm_{e}^{*}[s,L\left({\bar{p}}_{c}-{\bar{p}}_{r}\right)] & M_{cc}=\left(1-\rho \right)Nm \end{array} & \text{(9f)} \end{array}$$

Note that Eq (9f) is similar to Eq (8c), but with the effective migration rate $m_{e}\left[s,L\left({\bar{p}}_{c}-{\bar{p}}_{r}\right)\right]$ for selected alleles (Eq (4)) replaced by the corresponding rate $m_{e}^{*}\left[s,L\left({\bar{p}}_{c}-{\bar{p}}_{r}\right)\right]$ for neutral alleles (Eq (5)).

Since we assume equal and opposite selective effects of any allele in the two habitats (*s*_*c*_=*s*_*r*_=*s*), we have: (1−*ρ*)*M*_*rr*_=*ρM*_*cc*_ and (1−*ρ*)*M*_*cr*_=*ρM*_*rc*_ (Eq (9f)). From this, it also follows that $F_{ST}^{\left(rc\right)}=\left(F_{ST}^{\left(rr\right)}+F_{ST}^{\left(cc\right)}\right)/2$. However, this does not hold generally, e.g., with asymmetric selection across habitats.

### Individual-based simulations

Individual-based simulations are used to check the accuracy of the various approximations introduced above. In case of the mainland-island model, a single focal deme with *N* haploid individuals carrying *L* selected unlinked loci is simulated. In each generation, a Poisson number of individuals with mean *Nm* is replaced by mainland individuals, who carry the locally deleterious allele at each of their *L* loci. Mutation is then introduced by flipping the state of each locus of each individual independently with probability *μ*. Selection is implemented by multinomial sampling of 2*N* parents (with replacement) from the existing *N* individuals by choosing sampling weights to be equal to (relative) individual fitness. *N* offspring in the next generation are then produced by randomly pairing parents and creating offspring genotypes by independently choosing the allelic state at each locus to be the same as that of either parent with equal probability.

The simulation procedure for the island model with *D* islands is the same (excluding mutation), except that migration is implemented by first removing a Poisson-distributed number of individuals (with mean *Nm*) from each deme to create a common migrant pool; individuals from this pool are then randomly redistributed back into the *D* demes, while ensuring that the size of each deme remains constant at *N*. Simulations are initialized such that there is maximum polymorphism (allele frequency 0.5) at each locus within each population; other initial conditions are considered in Section 6 in S1 Text.

For the *D*-island model, an additional set of *L*_1_ neutral bi-allelic markers are simulated; these are unlinked to each other and to selected loci, and are also initialized with allele frequency 0.5. Once the population has equilibrated, the single-deme *F* statistics are calculated as: $F_{ST}^{\left(r\right)}=1-\pi_{W}^{\left(r\right)}/\pi_{T}$ and $F_{ST}^{\left(c\right)}=1-\pi_{W}^{\left(c\right)}/\pi_{T}$, where $\pi_{W}^{\left(r\right)}$ (respectively $\pi_{W}^{\left(c\right)}$) is the heterozygosity within demes in the rare (resp. common) habitat, averaged over all neutral markers and over all demes in the habitat; *π*_*T*_ is the diversity across the whole population, averaged over all neutral markers. Similarly, the 2-deme F measures are calculated as: $F_{ST}^{\left(rr\right)}=\left(\pi_{B}^{\left(rr\right)}-\pi_{W}^{\left(r\right)}\right)/\left(\pi_{B}^{\left(rr\right)}+\pi_{W}^{\left(r\right)}\right)$, $F_{ST}^{\left(cc\right)}=\left(\pi_{B}^{\left(cc\right)}-\pi_{W}^{\left(c\right)}\right)/\left(\pi_{B}^{\left(cc\right)}+\pi_{W}^{\left(c\right)}\right)$ and $F_{ST}^{\left(rc\right)}=\left(\pi_{B}^{\left(rc\right)}-\frac{\pi_{W}^{\left(r\right)}+\pi_{W}^{\left(c\right)}}{2}\right)/\left(\pi_{B}^{\left(rc\right)}+\frac{\pi_{W}^{\left(r\right)}+\pi_{W}^{\left(c\right)}}{2}\right)$. Here, $\pi_{B}^{\left(rr\right)}$, $\pi_{B}^{\left(cc\right)}$ and $\pi_{B}^{\left(rc\right)}$ denote the number of pairwise differences per site between 2 demes belonging to the same or different habitats; these are calculated (as above) by averaging over all neutral markers and over all relevant pairs of demes.

## Results

I first analyse the mainland-island model, focusing on how local adaptation on the island depends on *m*/*s* (the migration rate relative to per locus selection), *Ns* (the strength of selection per locus relative to drift), and *Ls* (the net selection against maximally deleterious genotypes; this is the main determinant of the magnitude of the barrier effect due to LD). Since the focus is on the interplay between migration, multi-locus selection and drift, the mutation rate is set to a fixed value throughout the main paper. The sensitivity of local adaptation thresholds to mutation is considered in Section 4 in S1 Text.

I then consider the infinite-island model, where there is an additional parameter *ρ* (which parametrizes the relative abundances of the two habitats). I focus on the conditions under which LD widens the range of migration rates over which local adaptation can be maintained in the rare habitat, and on the effect of polygenic barriers on neutral divergence between habitats. Throughout, theoretical predictions (Eqs (7)–(9)) using Eqs (4) and (5) are compared against individual-based simulations.

### Mainland-island model

**Fig 1:**
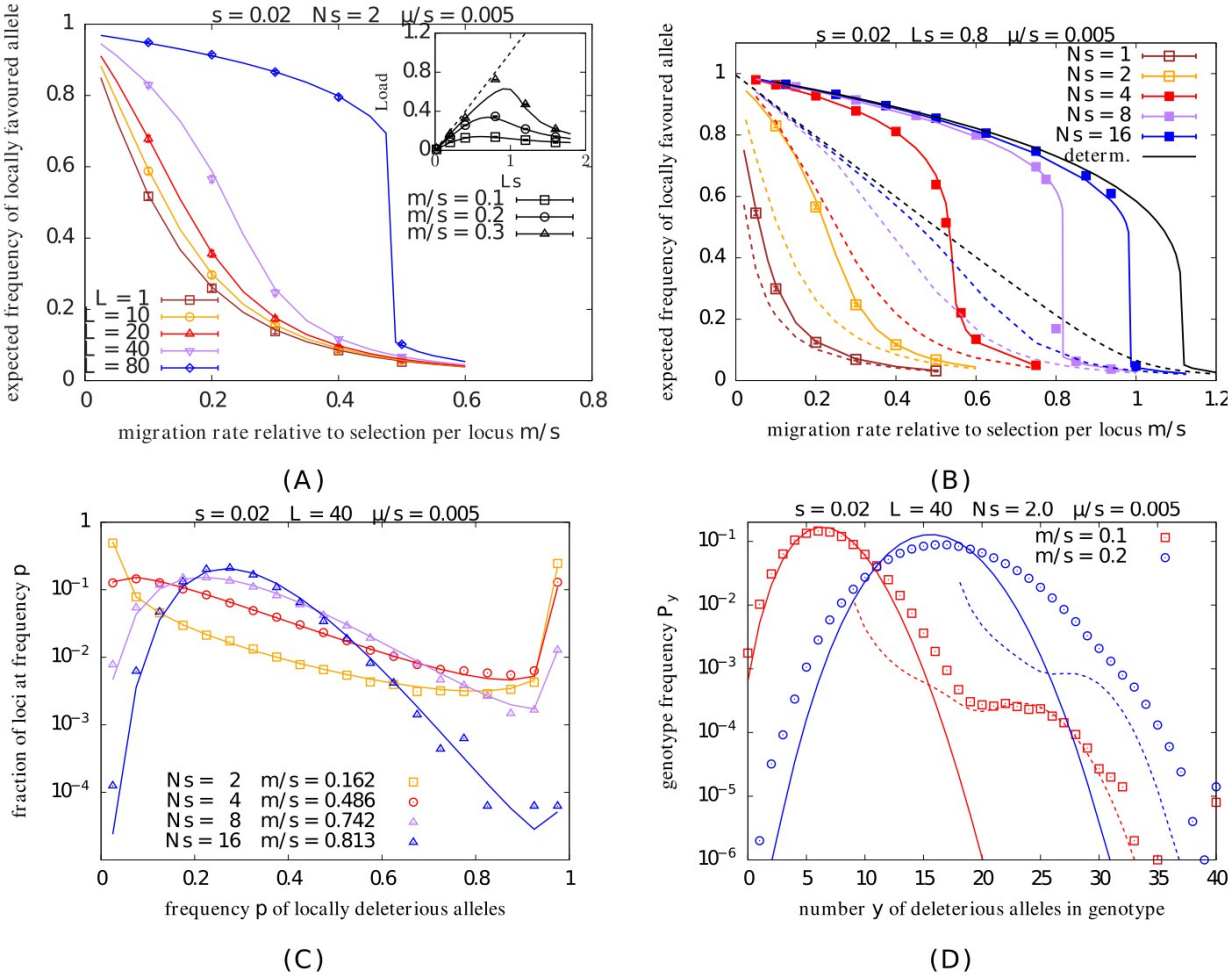
Local adaptation under mainland-island migration. A. Expected frequency 1-E[p] of the locally favoured allele on the island vs. *m*/*s*, the migration rate relative to selection per locus, for various *L* (different colors) for *s*=0.02, *Ns*=2, and *μ*/*s*=0.005. Inset: Expected load vs. *Ls* (which is varied by changing *L*) for various values of *m*/*s*. The maximum possible load *Ls* (dashed line) is also shown for reference. B. Expected frequency of the locally favoured allele vs. *m*/*s* for various *Ns* (different colors) for *s*=0.02, *L*=40 and *μ*/*s*=0.005. Symbols depict results of individual-based simulations in both Fig 1A and 1B (obtained by averaging over 100–200 simulation replicates for each point). Colored solid lines show theoretical predictions that account for both LD and drift (obtained from Eq (7) together with Eq (4)); colored dashed lines in 1B show LE/single-locus predictions that only account for drift (and are obtained from Eq (1)). Fig 1B also shows deterministic predictions that account for LD (solid black line) as well as the LE/single-locus deterministic prediction (dashed black line). See main text for how these are calculated. Note that there are no simulation results for the deterministic case (as individual-based simulations are always affected by drift). C. Distribution of allele frequencies shown by plotting the fraction of loci with frequency of locally deleterious allele between *p* and *p*+Δ*p*, vs. *p* (for Δ*p*=0.05). The different colors show distributions for *N*=100, 200, 400, 800 (which correspond to *Ns*=2, 4, 8, 16, for *s*=0.02), with *m*/*s* chosen in each case such that the expected frequency of the locally deleterious allele is 0.3. Theoretical allele frequency distributions (lines) match well with those from individual-based simulations (symbols), with some (moderate) deviation in larger populations. Theoretical predictions are obtained using Eq (1) with *m* replaced by *m*_*e*_, which depends on E[p], which is determined numerically, as above. D. Genotype frequencies *P*_*y*_, which represent the probability that a randomly chosen genotype in the population carries *y* deleterious alleles, vs. *y*, for two different values of *m*/*s*, for *Ns*=2 and *s*=0.02. Symbols depict results of individual-based simulations; dashed lines show deterministic predictions and solid lines predictions under LE (see text for more details about the two kinds of predictions). Other parameters for C. and D. are: *L*=40 and *μ*/*s*=0.005.

Fig 1A (main plot) shows the expected equilibrium frequency of the locally adaptive allele on the island as a function of *m*/*s*, for different numbers *L* of divergently selected loci (various colors), with the other parameters being *s*=0.02, *Ns*=2, *μ*/*s*=0.005. As expected, locally adaptive alleles become less common with increasing migration for any *L*. However, in contrast to the relatively smooth decrease in allele frequency with *m*/*s* observed for small *L*, there is a threshold effect when *L* is large—frequencies decrease only mildly with migration at low *m*/*s*, but then collapse beyond a critical migration threshold. Accordingly, adaptive allele frequencies can be much higher than the single-locus/LE prediction (shown in brown in Fig 1A), when *L* is large. This is simply a consequence of stronger multilocus selection against individuals with recent immigrant ancestry (at larger *Ls*), which causes sets of deleterious alleles to be eliminated together, before they can break up by backcrossing with fitter backgrounds.

The inset of Fig 1A shows the expected maladaptation load (calculated by summing across all loci) vs. *Ls*, where *Ls* is varied by varying *L*, while holding *s*=0.02 constant. The dashed line depicts the maximum possible load (also equal to *Ls* in this model): this corresponds to an extreme scenario where the island population is fixed for locally deleterious alleles at all loci. We see that load is actually highest for intermediate values of *Ls*, which can be understood as follows: at small *L*, any increase in the number of loci contributing to maladaptation far outweighs the milder decrease in deleterious allele frequency per locus. However, once *Ls* is large enough for selective elimination of groups of alleles to be effective, deleterious allele frequencies may decrease quite sharply with increasing *L*, which compensates for the increase in the number of loci, causing total load to decline with *L*.

Our focus on the composite parameter *Ls* is justified by the fact that effective migration rates depend primarily on *Ls*, rather than on *L* and *s* separately (see Eq (6a)). The sensitivity of allele frequencies to the exact genetic basis of load, i.e., to *L* and *s* (for a given *Ls* and *Ns*) is explored in Section 3 in S1 Text.

A striking feature of Fig 1A is the close agreement between the results of individual-based simulations (symbols) and theoretical predictions (lines), which are obtained by numerically iterating Eq (7). This rather successful approximation of the effects of LD on allele frequencies in terms of effective migration rates suggests the following explanation for sharp thresholds for loss of local adaptation at large *Ls*: a small increase in maladaptation at many loci may, in aggregate, cause a substantial increase in the effective migration rate $m_{e}\approx me^{-2Ls\left(1-E[p]\right)}$ associated with individual alleles, provided *Ls* is large. This, in turn, further increases swamping, pushing up frequencies of locally deleterious alleles, setting in motion a positive feedback, which culminates in collapse of local adaptation above a threshold migration rate.

Consider next how genetic drift influences local adaptation. Fig 1B shows the expected frequency of the locally favoured allele as a function of *m*/*s*, the migration rate relative to selection strength per locus, for island populations of different size (different colors). As before, simulation results (symbols) match theoretical predictions (colored solid curves) very well across all parameter combinations. Larger sizes (which correspond to higher values of *Ns*) allow populations to sustain local adaptation at significantly higher migration levels. For example, while the frequency of the locally adaptive allele drops below 0.1 already at *m*/*s*≈0.25 in the smallest population (with *Ns*=1), the corresponding threshold is *m*/*s*≈0.98 in the largest population (with *Ns*=16), close to the deterministic threshold *m*/*s*≈1.11. Here, the deterministic equilibrium allele frequency *p*_*det*_ at migration-selection-mutation balance is obtained by numerically solving: −*s p*_*det*_(1−*p*_*det*_)+*m*_*e*_[*s*, *L*(1−*p*_*det*_)](1−*p*_*det*_)+*μ*(1−2*p*_*det*_)=0, and using the approximate expression in Eq (6a) for *m*_*e*_. This prediction (shown via the solid black curve in Fig 1B) accounts for the effects of LD between introgressing deleterious alleles but neglects drift. A comparison of the finite *Ns* plots with the deterministic prediction shows that genetic drift has a significant effect on local adaptation for *Ns*≲10 in this example.

Fig 1B also shows LE/single-locus predictions for each *Ns* (colored dashed curves; obtained from Eq (1)): these account for the effects of genetic drift but neglect LD. The LE prediction for the deterministic allele frequency *p*_*det,LE*_, obtained by solving −*s* *p*_*det,LE*_(1−*p*_*det,LE*_)+*m*(1−*p*_*det,LE*_)+*μ*(1−2*p*_*det,LE*_)=0, is also shown for reference (black dashed curve). While the frequency of the locally favoured allele (symbols/solid lines) is higher than the corresponding LE prediction (dashed lines) at all *Ns*, this effect is stronger in larger populations, which also exhibit sharper thresholds for loss of local adaptation. This can be understood, as before, in terms of the effect of genetic drift on effective migration rates—larger populations are less likely to fix locally deleterious alleles and thus have lower load. Consequently, migrants from the mainland have lower relative fitness in these populations (all other parameters being equal), causing effective immigration rates to be also lower in larger populations, thus further protecting locally adaptive alleles from swamping.

At first glance, it is surprising that this simple heuristic based on introducing effective migration rates into the single-locus diffusion approximation should accurately predict the expected allele frequency across such a wide range of parameters, including in large populations, where one or other allele is not necessarily close to fixation. This behoves us to ask: how sensitive are these approximations to assumptions about the (U-)shape of the underlying allele frequency distribution? We can investigate this by contrasting frequency distributions in populations of different size (Fig 1C), choosing the migration rate in each case such that the expected deleterious allele frequency is ∼0.3 (based on the theoretical prediction), regardless of size.

As expected, the allele frequency distribution is U-shaped in smaller populations, but unimodal and peaked around E[p]∼0.3 in larger populations. Interestingly, theoretical predictions for the frequency distribution (lines) are quite accurate even in the two largest populations (purple and blue triangles in Fig 1C, corresponding respectively to *Nm*∼6 and *Nm*∼13), for which distributions of the selected allele deviate markedly from the canonical U-shape, and are characterised by high heterozygosity (E[2pq]∼0.365 and ∼0.399 respectively). Thus, this rather crude representation of multi-locus LD via a single effective migration rate, that depends only on the expected number of genetic differences between the mainland and island, appears to suffice even if there is substantial polymorphism, i.e., if these differences represent pairwise differences between individuals rather than fixed differences between populations. This is likely due to the fact that, to lowest order in *s*, the effective migration rate only depends on the average divergence between and not heterozygosity within populations (see Models and methods).

Finally, we ask: can the approximations introduced here predict the equilibrium *genotype* frequency distribution? Fig 1D shows the equilibrium frequencies *P*_*y*_ for genotypes carrying *y*=0, 1, 2, … deleterious alleles, for two values of *m*, as found in simulations (symbols), along with two kinds of analytical predictions (solid and dashed lines). The solid lines show the predicted genotype frequencies *under LE* given the expected deleterious allele frequency E[p] (which is determined as described above, using Eq (8)). Under LE, the allelic states at different loci are statistically uncorrelated, and the probability of genotypes with exactly *y* deleterious alleles is $P_{y}^{\left(LE\right)}=\left(\begin{array}{c} L\\ y \end{array}\right){\left(E\left[p\right]\right)}^{y}{\left(1-E\left[p\right]\right)}^{L-y}$.

We also compare against the *deterministic* prediction for *P*_*y*_ (dashed lines in Fig 1D) obtained by solving Eq (3) (see also eq. 1 in S1 Text): more specifically, the dashed lines show $P_{y-LE[p]}^{\left(det\right)}$ vs. *y*, where the deterministic frequencies *P*^(*det*)^ are calculated by assuming that the island is subject to divergent selection at L(1-E[p]) loci. Note that the relevant deterministic frequencies are $P_{y-LE[p]}^{\left(det\right)}$ and not $P_{y}^{\left(det\right)}$ as genotypes carrying *y* deleterious alleles have relative (Malthusian) fitness that is proportional to y-LE[p] (since the island is nearly fixed for LE[p] deleterious alleles, by definition).

At the lower migration level (red plot in Fig 1D), genotype frequencies *P*_*y*_ are close to the LE prediction (solid line) for small *y*, while they match the deterministic prediction (dashed line) for large *y*, with a crossover between the LE and deterministic regimes at intermediate *y*. This suggests that when migration is low and deleterious alleles correspondingly rare, the frequencies of genotypes with large numbers of deleterious alleles (large *y*) are governed by the (deterministic) balance between migration, selection and recombination, with recombination only serving to break down highly deleterious genotypes into smaller, less deleterious fragments, but rarely bringing together such fragments to reconstitute the more deleterious genotypes (this is tantamount to assuming that {*P*_*y*_} satisfy *linear* coupled equations, as in Eq (3)). In contrast, both roles of recombination—the splitting of more deleterious genotypes to generate the focal genotype and the reconstitution of the focal genotype via recombination between less deleterious genotypes, appear to play a role in shaping the frequencies of genotypes with low numbers of deleterious alleles (small *y*): this is reflected in the fact that *P*_*y*_ for small *y* are close to the LE prediction, which assumes that genotypes are random assortments of independently segregating alleles. We note that *P*_*y*_ are slightly elevated above the LE prediction for very small *y*, reflecting positive selection on such genotypes due to their higher relative fitness.

At the higher migration level (blue plot), the distribution does not exhibit two distinct regimes. In particular, at large *y*, the *P*_*y*_ are much higher than the deterministic prediction, suggesting that when migration is high and deleterious genotypes more common, mating events which bring together different sets of deleterious alleles to generate highly deleterious genotypes are also relevant; accounting for such events would introduce terms of the kind *P*_*y*_ *P*_*y*′_ in Eq (3)). Also, the actual distribution of *P*_*y*_ for smaller *y* is significantly wider than the LE prediction in this case, suggesting significant selection not just on individual alleles but also sets of alleles.

The fact that these approximations nevertheless accurately predict allele frequency distributions at high levels of migration suggests that the heuristic of effective migration rates captures the *gross* effects of LD on allele frequencies quite robustly, even when it does not provide a good handle on LD (or equivalently, on genotype frequencies) itself. As before, this is a reflection of the fact that effective migration rates are insensitive to within-deme genetic variance (to lowest order in *s*), and thus, are relatively insensitive to within-deme LD (since it only contributes to the variance within demes).

### Infinite-island model

Let us now consider adaptive divergence between habitats in the infinite-island setting, where a non-zero fraction *ρ* of islands belong to the rare habitat. In this case, both habitats influence each other via maladaptive gene flow. Thus, we must consider how allele frequencies in the two habitats co-evolve, instead of assuming the state of the more abundant habitat to be ‘fixed’ and independent of the rare habitat, as in the mainland-island case.

In the absence of mutation, there is a well-defined critical migration rate *m*_*c*_, such that locally adaptive alleles can segregate in the rare habitat for *m* < *m*_*c*_, but are eliminated for *m* > *m*_*c*_. In other words, above this threshold, no long-term adaptive divergence between habitats is possible—instead, alleles favoured in the common habitat fix across all islands, irrespective of habitat. Before analysing the effects of drift and LD on this critical threshold systematically, it is useful to consider a few examples.

**Fig 2:**
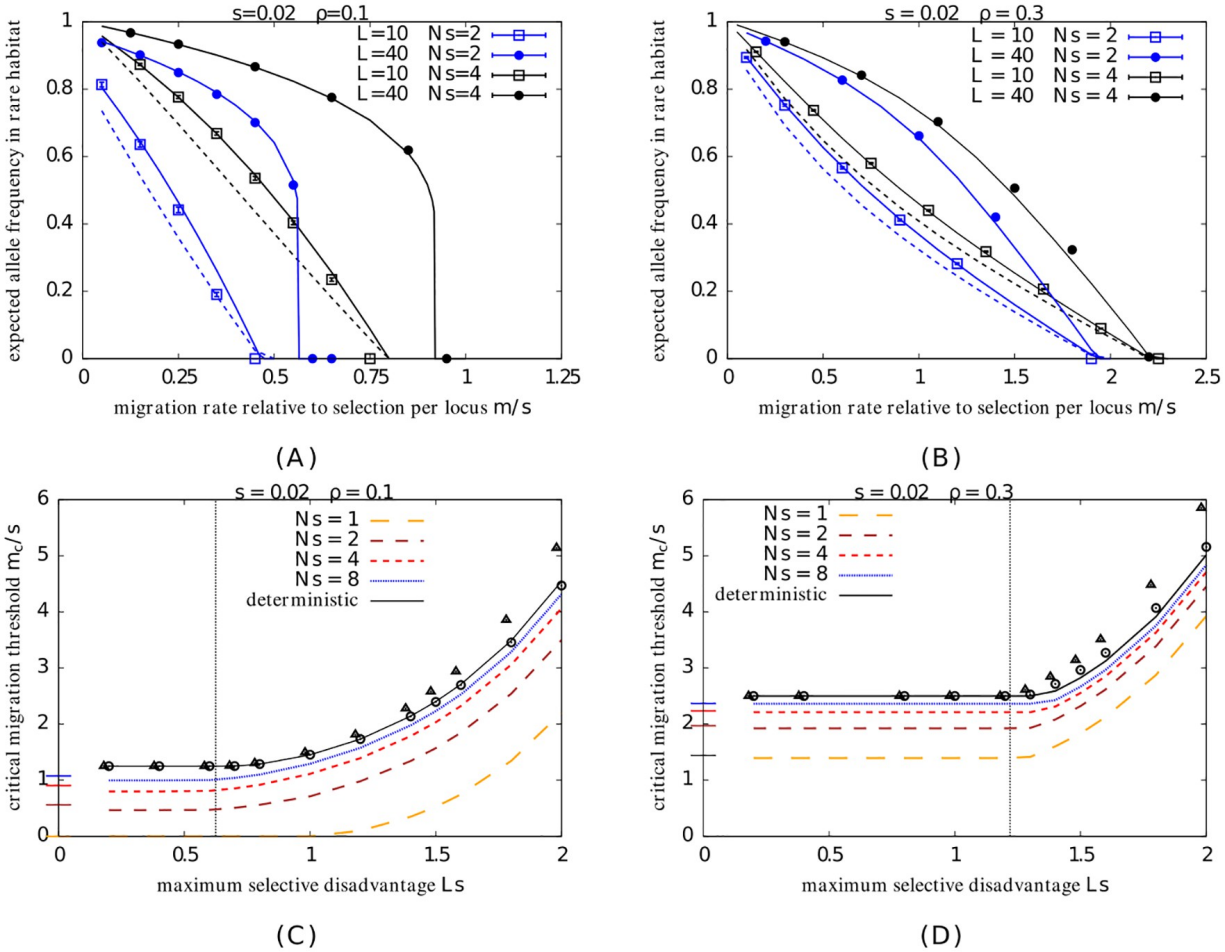
Local adaptation in the infinite-island model with two habitats. A–B. Expected equilibrium frequency of the locally adaptive allele in the rare habitat vs. *m*/*s* for *ρ*=0.1 (Fig 2A) and *ρ*=0.3 (Fig 2B), for 2 different values of *L* (10 and 40; squares vs. circles), and two different population sizes (corresponding to *Ns*=2 and *Ns*=4; blue vs. black). Symbols depict results of individual-based simulations; solid lines depict theoretical predictions that account for both LD and drift (obtained using Eq (8) together with Eq (4)); dashed lines depict LE (i.e., single-locus) predictions that only account for drift (obtained from Eq (2)). Selective effect per deleterious allele is *s*=0.02 in both plots. The number of simulated demes is *D*=500 in all individual-based simulations; the average allele frequency is obtained by averaging over all *L* loci and all *ρD* islands in the rare habitat, across 5 simulation replicates. C–D. Theoretical predictions for *m*_*c*_/*s*, the critical migration threshold scaled by the per-locus selection coefficient, vs. *Ls* for *ρ*=0.1 (Fig 2C.) and *ρ*=0.3 (Fig 2D.), for *s*=0.02 and *N*=50, 100, 200, 400 (corresponding to *Ns*=1, 2, 4, 8 respectively). Here, *m*_*c*_ is the critical migration threshold above which local adaptation cannot be maintained in the rare habitat. Theoretical predictions are obtained by solving for the polymorphic equilibrium of Eq (8) (using Eq (4)) and determining the value of *m* above which no such equilibrium exists. The short horizontal colored lines along the vertical axis represent the approximate LE (single-locus) prediction $\frac{m_{c}}{s}\approx \frac{1}{1-2\rho }\left(1-\frac{1}{2Ns}\text{log}[\frac{1-\rho }{\rho }]\right)$ (see [38]). The exact deterministic predictions for *m*_*c*_/*s* (obtained by solving coupled deterministic equations for *p*_*r*_ and *p*_*c*_; see eq. 12 in Section 5 in S1 Text) are shown using solid black lines. The critical migration rate *m*_*c*_/*s* is constant for small *Ls*, but then starts increasing with *Ls* beyond a threshold (*Ls*)_*_. The deterministic prediction for (*Ls*)_*_ is depicted by vertical dotted lines and depends only on the habitat fraction *ρ* (see text). In addition, we also show approximate deterministic predictions for *m*_*c*_/*s* (triangles and circles)—for *Ls*>(*Ls*)_*_ and in the highly polygenic limit *s* → 0, *L* → ∞ with *Ls* constant, the deterministic *m*_*c*_/*s* is given by Eq (10a) and is shown using triangles. Predictions that are more accurate at somewhat larger *s* are obtained in Section 5 in S1 Text (see eq. 17B in S1 Text). These are shown using circles and agree well with the numerically obtained deterministic *m*_*c*_/*s* (solid black lines).

Fig 2A and 2B show the expected frequency of the locally adaptive allele in the rare habitat, as a function of *m*/*s*, the migration rate relative to per-locus selection, for different *Ns* (blue vs. black colors), different *L* (different symbols in each subfigure), and different *ρ* (right vs. left columns). As before, theoretical predictions that account for both drift and LD (solid lines), obtained by numerically solving Eq (8) in conjunction with Eq (4), are in good agreement with the results of individual-based simulations (symbols) across all parameter combinations. LE/single-locus predictions that account for drift (but not LD) are also shown for reference (dashed lines).

Comparing Fig 2A and 2B, we see that the critical migration threshold is higher for larger *ρ*: thus, local adaptation in the rare habitat can be sustained over a wider range of migration rates when the relative abundances of the two habitats are more similar (so that a smaller fraction of genotypes immigrating into the rare habitat are deleterious). Further, *m*_*c*_/*s* also increases with *Ns*, especially for *ρ*=0.1 (blue vs. black plots in Fig 2A), suggesting that drift has a significant effect on local adaptation in rare habitats in this parameter regime.

For the parameters depicted here, the critical threshold is approximately the same for *L*=10 and *L*=40, and thus is insensitive to *Ls*, the total selection difference between habitats. As discussed below, this is only true if *Ls* is lower than a threshold (*Ls*)_*_, which depends on *ρ*. For *Ls*>(*Ls*)_*_, LD between locally adaptive alleles allows adaptation to be maintained over a much larger range of migration rates than would be possible for selection acting on one locus alone (see Fig 2C and 2D).

Note that higher values of *Ls* always result in *sharper* thresholds for loss of adaptation across all parameter combinations, even when the critical migration rate *m*_*c*_ (at which adaptation collapses) remains unchanged. As before, sharper thresholds are a consequence of stronger LD between loci at higher *Ls*. Thresholds are also sharper for smaller *ρ*, which can be rationalised as follows: sharp thresholds emerge when an increase in migration causes a substantial enough increase in load in the recipient population that the effective migration rate of deleterious alleles immigrating from the *alternative* habitat also rises significantly (due to a rise in the RV of migrants between habitats), setting in motion a positive feedback between declining population fitness and rising (effective) maladaptive immigration into the population. Note that this kind of positive feedback only involves alleles migrating between differently adapted habitats, and not deleterious alleles that migrate within the same habitat (see also Eq (8c)). Thus, feedback effects are stronger and the threshold for loss of local adaptation on an island within the rare habitat sharper if immigration into the island is predominantly from the alternative habitat (as is the case if *ρ* is small).

When does LD cause the critical migration threshold for loss of local adaptation to *shift* (in addition to becoming sharper), and to what extent are such shifts opposed by genetic drift? One can investigate this question systematically by plotting *m*_*c*_/*s* vs. *Ls* for various values of *N* (or equivalently, *Ns*, since *s*=0.02 is held constant). Here, *m*_*c*_ is the critical migration threshold above which no adaptive divergence is possible, regardless of the initial state of the population, *Ls* is a proxy for the (maximum possible) barrier effect due to LD, and 1/(*Ns*) measures the strength of genetic drift relative to selection per locus. Fig 2C and 2D show theoretical predictions for *m*_*c*_/*s* for various *Ns* (colored dashed lines) for *ρ*=0.1 and *ρ*=0.3 respectively. To disentangle the effects of LD and drift, it is useful to also consider the deterministic (*N*→∞) predictions for *m*_*c*_/*s* (shown by solid black lines). Details of the deterministic analysis are presented in Section 5 in S1 Text; only the main findings are summarised here.

#### Effect of LD on critical migration thresholds (neglecting drift)

The deterministic analysis identifies a threshold (*Ls*)_*_, above which LD between locally adaptive alleles is strong enough to raise the critical migration rate. The threshold (*Ls*)_*_ depends only on the relative abundances of the two habitats (in the deterministic limit), and is given by (*Ls*)_*_≈1/[2(1−2*ρ*)] for *ρ*≤1/4, and (*Ls*)_*_≈4*ρ* for 1/4<*ρ*<1/2 (see Section 5 in S1 Text). These thresholds are indicated by dotted vertical lines in Fig 2C and 2D.

For *Ls*<(*Ls*)_*_, there exists a single critical migration threshold, *m*_*c*,1_/*s*=1/(1−2*ρ*), which is independent of *Ls*. Both habitats evolve (partial) local adaptation for *m*<*m*_*c*,1_, resulting in non-zero allele frequency divergence between habitats at equilibrium. Conversely, no divergence can be maintained when *m*>*m*_*c*,1_, regardless of initial levels of divergence. Note that *m*_*c*,1_ is simply the threshold for the maintenance of polymorphism at deterministic migration-selection equilibrium for a *single* locus.

For *Ls*>(*Ls*)_*_, we observe two thresholds *m*_*c*,1_ (which is the LE/single-locus threshold for polymorphism, discussed above) and a second threshold *m*_*c*,2_>*m*_*c*,1_ (described below). There is always stable adaptive divergence between habitats at low migration rates, i.e., for *m*<*m*_*c*,1_, while migration necessarily erodes all adaptive divergence for *m*>*m*_*c*,2_ (even when the two habitats are initially perfectly locally adapted). At intermediate migration rates, i.e., for *m*_*c*,1_<*m*<*m*_*c*,2_, evolutionary outcomes depend on the *initial* state of the metapopulation. In particular, long-term divergence is possible either if initial divergence between habitats is high (as in a scenario of secondary contact between subpopulations that have diverged in allopatry) or if the rare habitat harbours sufficient adaptive variation that multilocus divergence can build up and LD-mediated barrier effects emerge faster than migration washes out allele frequency differences at individual loci.

Note that the rising part of the black curves in Fig 2C and 2D corresponds to *m*_*c*,2_ since the plots show critical migration thresholds above which long-term divergence is not possible, regardless of the initial state of the population.

The deterministic predictions for *m*_*c*_/*s* shown in Fig 2C and 2D (solid black lines) are obtained by numerically solving the deterministic equations for *p*_*c*_ and *p*_*r*_ (see eq. 13 in Section 5 in S1 Text). However, an approximate analytical expression for $m_{c,2}$ can be obtained in the limit of highly polygenic divergence (i.e., for *s* → 0, *L* → ∞ with *Ls* constant). This expression is given by Eq (10a) below and is shown using triangles in Fig 2C and 2D. Moreover, in this limit, we can also obtain the critical allele frequency divergence Δ_*c*_=*p*_*c*_−*p*_*r*_ between habitats, as *m* approaches *m*_*c*,2_ (see Eq (10b)).

$$\begin{array}{cc} \frac{m_{c,2}}{s}\approx \frac{Ls-2\rho -\sqrt{Ls\left(Ls-4\rho \right)}}{4\rho^{2}}\text{exp}[\frac{Ls\left(4\rho -1\right)+\sqrt{Ls\left(Ls-4\rho \right)}}{2\rho }] & \text{(10a)}\\ \Delta_{c}\approx \frac{Ls\left(4\rho -1\right)+\sqrt{Ls\left(Ls-4\rho \right)}}{4Ls\rho } & \text{(10b)} \end{array}$$

Thus, allele frequency divergence between habitats must be at least Δ_*c*_ for LD between adaptive alleles to maintain local adaptation. Once divergence falls below this level, there is a positive feedback between an increase in maladaptation load in the rare habitat and a corresponding increase in effective migration rate of deleterious alleles from the common to the rare habitat, leading to a sharp collapse in adaptive divergence.

#### Effect of drift (and LD) on critical migration thresholds

Let us now consider critical migration thresholds in populations where *Ns* is not too large and drift has significant effects on single-locus polymorphism. These thresholds are depicted by the various colored dashed curves in Fig 2C and 2D, and are obtained by numerically finding the migration rate above which no polymorphic fixed point (with $0<{\bar{p}}_{r}<1$) of Eq (8) exists.

As in the deterministic limit, *m*_*c*_/*s* is independent of *Ls* for small *Ls*, and is equal to the single-locus (LE) threshold, which is approximately: $\frac{m_{c}}{s}\approx \frac{1}{1-2\rho }\left(1-\frac{1}{2Ns}\text{log}[\frac{1-\rho }{\rho }]\right)$ for *ρ* not too small [38]. These single-locus predictions (with drift) are indicated by horizontal colored dashes along the vertical axis in Fig 2C and 2D. Thus, in this regime, drift reduces the critical migration threshold *m*_*c*_/*s* by an amount proportional to 1/(*Ns*), with the reduction being more significant when one habitat is much rarer than the other (i.e., for smaller *ρ*). In fact, adaptive alleles are necessarily lost from the rare habitat in the absence of LD-mediated effects if $\rho <\frac{1}{1+e^{2Ns}}$, or equivalently, $Ns<\frac{1}{2}\text{log}\left(\frac{1-\rho }{\rho }\right)$, *regardless* of migration level (see e.g., *Ns*=1 curve in Fig 2C).

As before, sufficiently strong LD may raise the critical migration rate *m*_*c*_/*s* above the single-locus threshold. This requires *Ls*>(*Ls*)_*_, where the threshold (*Ls*)_*_ appears to be insensitive to *Ns*, and is thus predicted by the deterministic analysis (dashed vertical lines in Fig 2C and 2D; see also above). Interestingly, even with $Ns<\frac{1}{2}\text{log}\;\left(\frac{1-\rho }{\rho }\right)$, i.e., when selection on individual alleles is too weak to counter *any* level of gene flow in the long run, LD between many such alleles can maintain local adaptation under moderate levels of migration—this is, for example, the case for *Ns*=1 in Fig 2C.

Note that the rising part of the *m*_*c*_/*s* vs. *Ls* curves has approximately the same shape in populations with large *Ns* (e.g., blue and red dashed curves) as in the deterministic case (black solid curve), and is merely shifted downwards with respect to the deterministic prediction. However, at smaller *Ns*, the shape is also affected, with *m*_*c*_/*s* rising more slowly with *Ls* than in larger populations. These effects are, however, rather modest, suggesting that the main effect of drift is to cause a fixed reduction (which is more significant for smaller *Ns* and smaller *ρ*) in the critical migration threshold *m*_*c*_/*s*, regardless of *Ls*.

As in the deterministic case, whether or not polygenic local adaptation can evolve despite high levels of migration (via the emergence of strong multilocus barriers to gene flow) depends on the initial level of adaptive variation available, especially in the rare habitat. Section 6 in S1 Text explores the behaviour of individual-based simulations initialized with different allele frequencies, for a few representative parameter combinations. With low initial adaptive variation in the rare habitat, populations may not be able to evolve enough divergence for LD-mediated effects to come into play. Then critical migration thresholds are lower than those depicted in the rising part of the *m*_*c*_/*s* vs. *Ls* curves in Fig 2C and 2D (though they may still be higher than the corresponding single-locus threshold, depending on the level of initial variation; see Section 6 in S1 Text).

#### Barriers to gene flow and neutral divergence

**Fig 3:**
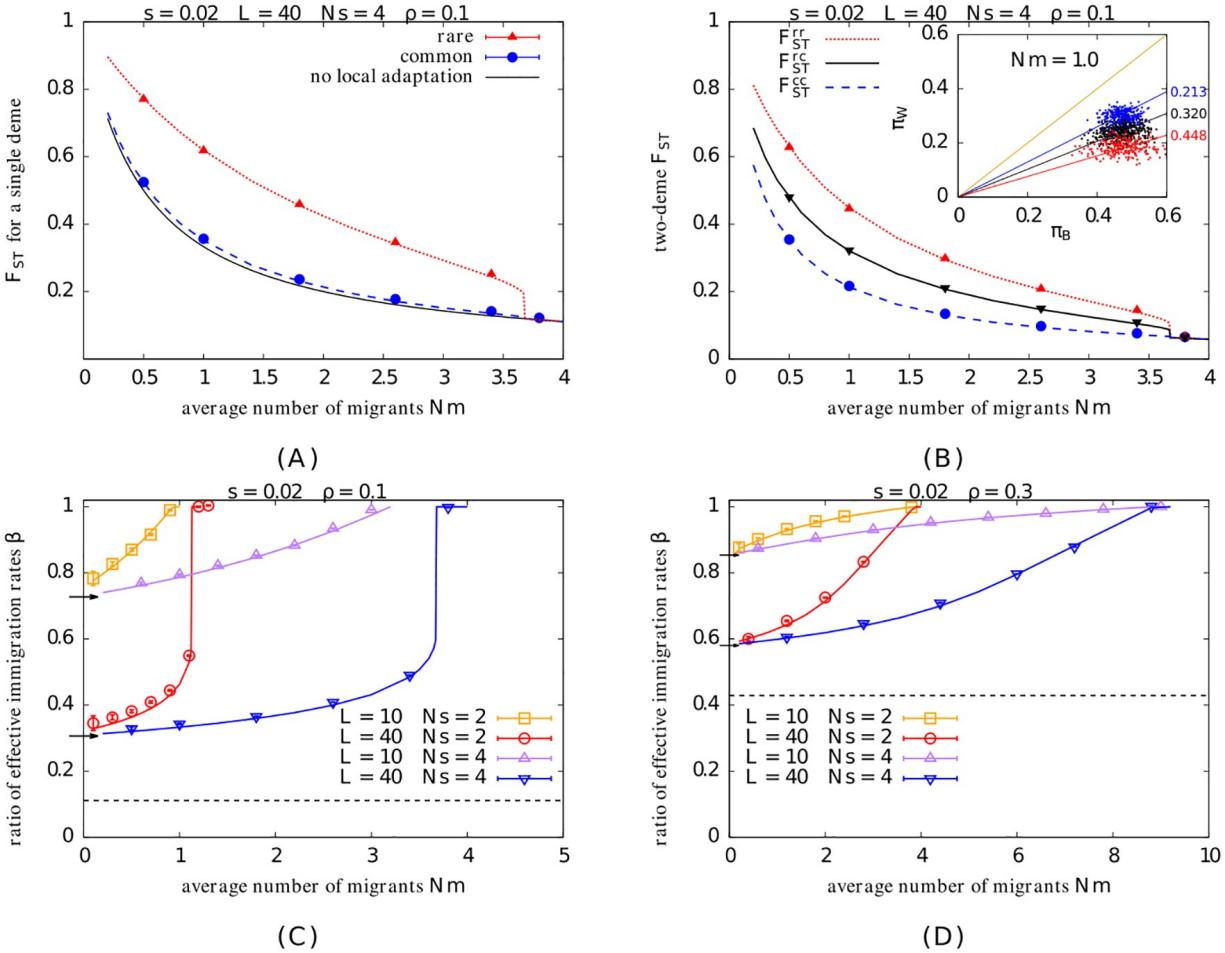
Neutral divergence in the infinite-island model. A. Average *F*_*ST*_ for a *single* deme in the rare $\left(F_{ST}^{\left(r\right)}\right)$ and common $\left(F_{ST}^{\left(c\right)}\right)$ habitats vs. *Nm*, for *s*=0.02, *L*=40, *Ns*=4, *ρ*=0.1. Here, *F*_*ST*_ is measured relative to the whole metapopulation. Symbols show results of individual-based simulations; dashed lines represent theoretical predictions (obtained from Eq (9a), (9b) and (9f) and using Eq (5)); the solid black line represents *F*_*ST*_=1/(1+2*Nm*)– the prediction in the absence of local adaptation. B. Average *F*_*ST*_ for a *pair* of demes vs. *Nm*, for the same parameters as in Fig 3A. Here, both demes within the pair may belong to the rare habitat ($F_{ST}^{\left(rr\right)}$), or both to the common habitat ($F_{ST}^{\left(cc\right)}$), or one to the rare and the other to the common habitat ($F_{ST}^{\left(rc\right)}$). The plots show simulation results (symbols) as well as theoretical predictions (lines; obtained from Eq (9c)–(9f) and using Eq (5)). *Inset* (Fig 3B): The neutral diversity within demes *π*_*W*_ vs. neutral divergence between demes $\pi_{B}$ for rare/rare, common/common and rare/common pairs of demes (shown using red, blue and black points respectively), for *Nm*=1.0, as measured at a single timepoint in an individual-based simulation. Each point represents a pair of demes (*i*, *j*); *π*_*W*_ is computed as $1/40\sum_{k=1}^{40}\left(2p_{i,k}q_{i,k}+2p_{j,k}q_{j,k}\right)/2$ and *π*_*B*_ as $1/40\sum_{k=1}^{40}\left(2p_{i,k}q_{j,k}+2p_{j,k}q_{i,k}\right)/2$, where *p*_*i*,*k*_ represents the allele frequency at the *k*_*th*_ neutral locus in deme *i* and *q*_*i*,*k*_=1−*p*_*i*,*k*_. The solid lines represent (*π*_*W*_, *π*_*B*_) combinations that would correspond to *F*_*ST*_ values of 0 (orange), 0.213 (blue), 0.320 (black), 0.448 (red): the last three are the predicted $F_{ST}^{\left(cc\right)}$, $F_{ST}^{\left(cr\right)}$ and $F_{ST}^{\left(rr\right)}$ at *Nm*=1 respectively. C– D. $\beta =\frac{\left(1/F_{ST}^{\left(r\right)}\right)-1}{\left(1/F_{ST}^{\left(c\right)}\right)-1}$ vs. *Nm* for *ρ*=0.1 (Fig 3C) and *ρ*=0.3 (Fig 3D), for different values of *L* and *Ns* (depicted by the different colors) and *s*=0.02. The quantity *β* gives the ratio of the *effective* number of immigrants per unit time into an island in the rare habitat to the corresponding number for an island in the common habitat. Theoretical predictions (solid lines) match simulation results (symbols) across all parameter combinations. The black dashed line in each plot represents the threshold *β*_*min*_=*ρ*/(1−*ρ*), which is the expected *β* under complete RI (wherein immigrants from the dissimilar habitat have zero RV). The short horizontal arrows along the vertical axis represent the threshold *β*=(*β*_*min*_+ *e*^−2*Ls*^)/(1+*β*_*min*_ *e*^−2*Ls*^) for *L*=10 (upper arrow) and *L*=40 (lower arrow). This is the expected value of *β* when allele frequency divergence between habitats is maximum (see text). *F*_*ST*_ values in simulations are computed from 40 unlinked neutral loci.

Let us now consider how adaptive divergence influences genetic diversity at unlinked *neutral* loci. In the absence of such divergence, *F*_*ST*_ depends only on *Nm*, the average number of migrants exchanged per generation between any island and the full population. Here, *Nm* may be viewed as a proxy for the physical subdivision of the population, which affects all subpopulations equally (under the island model). However, if habitats are locally adapted, then *F*_*ST*_ depends not only on the number of migrants exchanged but also on the RV of migrants, i.e., their long-term contribution to the neutral gene pool of the receiving population. As shown in this section (Fig 3), this dependency is captured accurately by expressing *F*_*ST*_ in terms of the elements of an effective migration rate matrix for neutral alleles (Eq (9)) and approximating effective migration rates as in Eq (5).

The rationale for focusing on *unlinked* neutral markers is that reduced gene flow at such markers signals the emergence of genome-wide, as opposed to localized, barriers to gene flow, and is thus a more appropriate measure of RI between habitats. Moreover, the barrier effect at *any* neutral site (even those in the vicinity of a particular barrier locus) is primarily due to unlinked loci, as long as selection acts on very many alleles of weak effect spread across a long genetic map [13].

Fig 3A shows average $F_{ST}^{\left(r\right)}$ and $F_{ST}^{\left(c\right)}$ for islands belonging respectively to the rare and common habitat, as a function of *Nm*. The expected *F*_*ST*_ in the absence of local adaptation, which is 1/(1+2*Nm*) for haploids, is also shown (solid black line). As before, theoretical predictions (lines, obtained from Eq (9a), (9b) and (9f), in conjunction with Eq (5)) are in close agreement with simulations (symbols). Adaptive divergence between habitats increases neutral *F*_*ST*_ in both habitats above the neutral expectation (solid line), but more so in the rare habitat. This can be understood by noting that $F_{ST}^{\left(r\right)}$ and $F_{ST}^{\left(c\right)}$ measure respectively the extent to which neutral diversity within any deme belonging to the rare or common habitat is reduced relative to gene diversity at the level of the population as a whole; this in turn depends on the effective rate of immigration into the deme. When both habitats are locally adapted, the majority (i.e., a fraction 1−*ρ*) of immigrants into the rare habitat have very low RV, as they originate from the common habitat and thus carry genotypes that are locally deleterious. By contrast, only a minority (i.e., a fraction *ρ*) of immigrants into the common habitat have low RV. This results in a stronger reduction in neutral diversity and concomitantly, a sharper increase in *F*_*ST*_ for islands in the rare as compared to the common habitat.

We can also measure *F*_*ST*_ between pairs of islands in the population. Fig 3B (main plot) shows theoretical predictions (lines) and simulation results (symbols) for average $F_{ST}^{\left(rr\right)}$, $F_{ST}^{\left(rc\right)}$ and $F_{ST}^{\left(cc\right)}$, which correspond respectively to two islands both belonging to the rare habitat, or one to the rare and the other to the common habitat, or both to the common habitat. Note that $F_{ST}^{\left(rr\right)}>F_{ST}^{\left(rc\right)}>F_{ST}^{\left(cc\right)}$: thus, at low *Nm*, *F*_*ST*_ between two islands in the rare habitat is *greater* than *F*_*ST*_ between islands from different habitats, despite the fact that only the latter exhibit adaptive divergence.

This somewhat paradoxical observation is explained by the fact that the rare habitat, though rare, is nevertheless large enough to harbour high genetic diversity (at the level of the habitat as a whole), as long as it encompasses a finite fraction of all islands in a large metapopulation. Thus, any two islands within the rare habitat exhibit high neutral divergence with respect to each other (since they will each be close to fixing a random set of alleles segregating in the habitat as a whole), even as the diversity within each is strongly reduced because of very low net effective immigration. By contrast, islands that support the common habitat have fairly high within-island diversity as well as between-island divergence, causing *F*_*ST*_ to be lower for any pairwise comparison that includes such islands. This is illustrated in the inset of Fig 3B by plotting neutral diversity within demes, *π*_*W*_, vs. neutral divergence between demes, *π*_*B*_, for *Nm*=1, for pairs of demes belonging to the same vs. different habitats (different colors). This plot shows that differences in *F*_*ST*_ are purely due to differences in *π*_*W*_, as *π*_*B*_ is the same for any pair of demes (on average), irrespective of habitat.

Thus, different levels of *F*_*ST*_ for islands within the rare vs. common habitat reflect the underlying asymmetry in the effective levels of immigration into the two kinds of islands when both are at least partially locally adapted. This asymmetry, in turn, is due to the fact that the majority of immigrants into the rare habitat have low RV, while this is true of only a minority of immigrants into the common habitat. This asymmetry can be measured directly via $\beta =\frac{\left(1/F_{ST}^{\left(r\right)}\right)-1}{\left(1/F_{ST}^{\left(c\right)}\right)-1}$, which is the ratio of the effective number of immigrants per generation into islands within the rare habitat to the corresponding number for islands in the common habitat.

In the absence of local adaptation (i.e., if alleles favoured in the common habitat are fixed across the entire population at all divergently selected loci), the relative fitness of all immigrants between and within habitats is equal to that of residents for any island. Then, the effective number of immigrants into any island is equal to the actual number, irrespective of habitat (at least under soft selection), so that *β*=1. In the opposite limit of complete RI, individuals migrating between habitats have RV close to zero, while migrants within a habitat have RV close to 1. Then, the effective number of immigrants into any island is the same as the number of immigrants that originate from other islands within the same habitat, so that $\beta =\beta_{min}=\frac{\rho }{1-\rho }$.

Fig 3C and 3D show *β* vs. *Nm* for *ρ*=0.1 (Fig 3C) and *ρ*=0.3 (Fig 3D), for various values of *Ns* and *L* (same as those in Fig 2). As before, symbols represent results of individual-based simulations, while lines represent theoretical predictions; the horizontal dashed lines represent *β*_*min*_, which is the expectation under complete RI. As expected, *β* decreases as *Nm* decreases due to the concomitant increase in adaptive divergence between habitats. However, *β* always lies above the threshold *β*_*min*_ (dashed line) even for the smallest *Nm* that we simulate, indicating that RI is *incomplete*, even at very low levels of migration, as long as *Ls* is not very large. More precisely, we have: $\beta \approx \frac{\rho +\left(1-\rho \right)e^{-2Ls\Delta }}{\rho e^{-2Ls\Delta }+1-\rho }$, where $\Delta ={\bar{p}}_{c}-{\bar{p}}_{r}$ is the allele frequency divergence between two habitats, and *e*^−2*sL*Δ^ the RV of migrants between habitats. Thus, even with complete allele frequency divergence (Δ≈1), *β* will be only be (*β*_*min*_+*e*^−2*Ls*^)/(1+*β*_*min*_ *e*^−2*Ls*^), which is depicted by short horizontal lines along the vertical axis in Fig 3C and 3D.

Note that the typical values of *F*_*ST*_ found here—$F_{ST}^{\left(rc\right)}$ in the range 0.2−0.6 for *Nm*<2 (Fig 3B), correspond to the so-called “grey zone” of speciation, where it may be difficult to estimate effective migration rates and the extent of RI from sequence data using standard demographic inference methods [44]. The analysis here suggests that such values of *F*_*ST*_ are consistent with incomplete RI (and thus low levels of ongoing gene flow between habitats), at least in the context of patchy populations.

## Discussion

This paper introduces a simple heuristic for approximating the combined effects of LD and genetic drift on allele frequencies, when multiple loci are under divergent selection across distinct habitats in a subdivided population. It thus extends previous theory on polygenic barriers to gene flow (e.g., [5], [13]) to account for the effects of genetic drift within sub-populations. Drift may be significant when barrier loci have modest effects individually and/or when the scale of density regulation in populations is sufficiently local that *Ns* is small. However, divergent selection involving many such loci (large *Ls*) may still allow for local adaptation and substantial genomewide divergence between habitats, if migration levels are below a critical threshold.

### Effect of LD on critical migration thresholds for loss of local adaptation

A key finding of this study is that LD between adaptive alleles increases the critical migration threshold for loss of local adaptation only if *Ls* (which governs the strength of divergent selection) is above a threshold (*Ls*)_*_. This threshold depends primarily on the relative proportions of the two habitats in the population (neglecting drift): for instance, when the rare habitat encompasses 20% of demes (*ρ*=0.2), we have (*Ls*)_*_ ≈ 0.83, which corresponds to a relative immigrant fitness of $e^{-Ls_{*}}\approx 0.43$ under conditions of very low gene flow (i.e., when alternative alleles are fixed at all *L* loci across habitats).

Below this threshold, i.e., for *Ls*<(*Ls*)_*_, LD is not strong enough to maintain local adaptation beyond the single-locus migration threshold *m*_*c*,1_, though it does increase the extent of adaptive divergence between habitats for *m*<*m*_*c,1*_. By contrast, with *Ls*>(*Ls*)_*_, LD between alleles can maintain adaptive divergence at migration levels that may be several times higher than the *m*_*c*,1_ (see Fig 2C and 2D). The protective effects of LD on local adaptation are especially marked in very rare habitats, allowing these to withstand comparable levels of maladaptive migration as habitats that encompass a larger fraction (though still a minority) of islands. For example, in the absence of LD, the deterministic single-locus threshold for maintenance of adaptation in the rare habitat (*m*_*c*,1_/*s*=1/(1−2*ρ*)) is 1.25 for *ρ*=0.1 and 2.5 for *ρ*=0.3 (i.e., greater by a factor of 2 for the larger *ρ* value). However, at *Ls*=1.5, i.e., with LD, the corresponding deterministic thresholds (given by Eq (10a); see also Section 5 in S1 Text) are ≈2.6 for *ρ*=0.1 and ≈3.2 for *ρ*=0.3, (i.e., greater by a factor of only 1.2).

An important caveat of this analysis is that we assume that there is always enough standing genetic variation to allow for a rapid buildup of initial divergence across habitats (though see Section 6 in S1 Text for some examples with limited initial variation). This is then further reinforced by the positive feedback between increasing divergence and falling effective migration rates, provided *Ls*>(*Ls*)_*_ and migration is below a certain threshold. While rapid adaptation from standing genetic variation (possibly within a hybrid swarm) has been implicated in adaptive radiations [45, 46], this is hardly the norm. More generally, if adaptive divergence is mutation-limited to at least some degree, then initial divergence would build up slowly and may be seeded by pre-existing adaptive differences (so-called divergence hitchhiking [14]), until net divergence reaches a high enough value that genomewide effective migration rates drop, causing genomes in the different populations to ‘congeal’ [21, 22].

Here, we identify a related threshold: if allele frequency divergence falls below Δ_*c*_, there is a sharp increase in the genomewide effective migration rate, and divergence collapses. In the previous example with *ρ*=0.2 and assuming *Ls*=1, allele frequency divergence between habitats must be at least ≈0.3, i.e., migrant fitness no greater than 0.73 relative to population mean fitness and *m*_*e*_/*m* no greater than 0.54, for congealing to be possible, and for genome-wide barriers to persist at migration levels that would swamp individual adaptive alleles. An interesting question is how thresholds for congealing depend on the history of diverging populations, and whether certain population histories (e.g., involving rapid polygenic adaptation from standing genetic variation, as considered here) may lead to the emergence of genomewide congealing and RI over much shorter timescales than in the mutation-limited case (see also [47] for a discussion of rapid evolution of RI).

For local adaptation to be possible despite high migration (i.e., beyond the single-locus critical migration threshold) in our model, allele frequency divergence per locus must be significant (i.e., *p*_*c*_−*p*_*r*_ > Δ_*c*_): this is a consequence of the rather extreme form of divergent selection, involving multiplicative fitness costs across loci, that we consider. In a more realistic setting with stabilizing selection towards different optima across different habitats, substantial divergence at the level of the quantitative trait may evolve even with very little differentiation at the underlying trait loci [25, 26, 48]. It is less clear whether the positive feedback between increasing trait divergence and falling effective migration rates that is so prominent under multiplicative selection also plays a role in this case, and how this depends on the genetic architecture of traits. Extending the approximations developed here to the case of stabilizing selection thus remains an interesting direction for future work.

### Effect of genetic drift on critical migration thresholds

Critical migration thresholds may be significantly reduced by drift when the loci underlying local adaptation have modest (*Ns*≲10) effects (see, e.g., Figs 1B, 2C and 2D; also [23]), and when one habitat is much rarer than the other (*ρ* small). Moreover, the magnitude of this reduction is not very sensitive to *Ls*, with drift depressing critical migration thresholds by roughly similar amounts in both the LD-independent and the LD-dominated parameter regimes (i.e., for both the constant as well as the rising parts of the curves in Fig 2C and 2D).

Since direct estimation of the effect sizes of loci underlying adaptive divergence is difficult, one might turn the argument around and ask: given typical estimates of migration– between 1 and 10 ‘effective’ number of migrants exchanged per generation (see e.g., [49]), how strongly selected would a locus need to be to sustain adaptive divergence in the face of gene flow? Assuming *ρ*=0.1 in our model, adaptive divergence at a single locus requires *Ns*>9.7 with *Nm*=10 and *Ns*>2.05 with *Nm*=1. One might also look at an example where there is a modest genomewide barrier effect—with the same habitat proportions (*ρ*=0.1), *L*=100 and *s*=0.01 (so that *Ls*=1), we require *Ns*>7.6 with *Nm*=10 and *Ns*>1.7 with *Nm*=1 for adaptive divergence. The fact that the threshold *Ns* only decreases by a factor of ≲5 when *Nm* decreases by a factor of 10 (so that the threshold *m*/*s* is lower at lower *Nm*) is a consequence of genetic drift.

More broadly, this suggests that even though the effect sizes of loci contributing to local adaptation will depend on the size *N* of local demes, the *shape* of the distribution of effect sizes depends only on the rate of local drift relative to migration, i.e., on *Nm*. Thus, if smaller demes are connected by higher levels of migration, so that *Nm* is roughly similar across populations with very different local deme sizes, then critical thresholds and the shape of the effect size distribution (on the *s*/*m* scale) would also be very similar across populations. Conversely, if smaller demes are also more cutoff from each other on average (i.e., have lower *Nm*), then the distribution of adaptive differences should be biased towards larger effect loci (on the *s*/*m* scale) in populations with smaller local demes.

### Effect of adaptive divergence on genome-wide neutral *F*_*ST*_

A striking observation that emerges from the analysis of the infinite-island model is that neutral, genomewide *F*_*ST*_ is actually highest for a pair of subpopulations belonging to the rare habitat (Fig 3B), even though there is *no* RI (and no isolation-by-distance) between them. This counter-intuitive finding can be rationalised by noting that *F*_*ST*_ between a pair of demes (within a larger mosaic of interconnected demes) does not measure the extent of *direct* genetic exchange between them, but instead depends on the level of exchange between an individual deme and the whole population (averaged over the two demes). Since demes in the rare habitat are more effectively isolated from the larger population than those in the common habitat, it follows that the average level of genetic exchange between any two randomly chosen demes and the population as a whole is lowest if both demes are in the rare habitat, and highest if both are in the common habitat, so that $F_{ST}^{\left(rr\right)}>F_{ST}^{\left(rc\right)},>F_{ST}^{\left(cc\right)}$ when habitats are at least partially locally adapted.

The fact that neutral diversity in the rare habitat is reduced much more strongly than in the common habitat is, to some extent, a consequence of the infinite-island setting, in which individuals are assumed to migrate from any island to any other with equal probability, irrespective of their habitat of origin or destination. One could consider alternative models, where islands are embedded in a 2D spatial matrix, and where habitats are spatially sorted, so that migration between islands belonging to the same habitat is more likely than between islands belonging to different habitats (models with habitat choice but no explicit space might result in qualitatively similar outcomes). However, as long as spatial sorting of habitats and/or habitat choice is not complete, we expect effective immigration into the rare habitat to be at least somewhat lower than into the common habitat, and average *F*_*ST*_ between demes belonging to the rare habitat to be accordingly highest, despite the lack of RI between them.

In their re-analysis of so-called genomic islands of divergence across various hybridizing populations, Cruikshank and Hahn (2014) [50] pointed out that increased *F*_*ST*_ may reflect reduced diversity (e.g., due to purifying selection within populations) rather than increased divergence (due to reduced gene flow between populations). A consideration of local adaptation in extended populations yields yet another caveat: where sampled subpopulations are embedded within a larger metapopulation, reduced gene flow between subpopulations belonging to different ecological niches can manifest itself primarily via reduced diversity within, rather than increased divergence between, subpopulations (inset, Fig 3B). Moreover, this reduction is more severe for subpopulations which support the niche that is more marginal or less abundant in the population as a whole. While this effect would be less extreme in reality than it is under the infinite-island model, it nevertheless points to the perils of neglecting the wider spatial context of two (or a few) sampled subpopulations when interpreting relative divergence between them. Note that this caveat also applies to markers linked to barrier loci (since the underlying argument is based only on there being a rare and a common habitat), and thus is also relevant to the interpretation of solitary peaks of *F*_*ST*_.

### Approximating the effects of LD via effective migration rates

The theoretical approximations developed here rely on two kinds of separation of timescales. First, we assume that immigrant genomes split up (via recombination) over timescales that are much shorter than those associated with the evolutionary dynamics of individual loci. Then LD *within* any subpopulation is weak, even when there is substantial LD at the level of the metapopulation as a whole. Thus, we can use single-locus (diffusion) results for allele frequencies, while approximating the effects of metapopulation-wide LD through an effective migration rate. In general, we expect this assumption to be valid if loci are unlinked or possibly weakly linked (and as long as *s*, *m*, 1/*N* ≪ 1). By contrast, if deleterious alleles are tightly linked over a block of genome, then the effective unit of selection is the full block (rather than individual loci), and allele frequencies are shaped essentially by the balance between *m* and *Ls* [5].

Another factor that could affect the separation between timescales associated with single-locus and multi-locus dynamics is epistasis. For a given *F*_1_ fitness, diminishing returns epistasis between deleterious alleles (wherein selection per deleterious allele is weaker when many such alleles act in combination) tends to further strengthen the barrier to gene flow. Conversely, synergistic epistasis (alleles more deleterious in combination), which emerges naturally in models of stabilizing selection on additive traits, weakens the barrier relative to multiplicative selection [13]. While the prevalence of diminishing-returns epistasis in nature is unclear [51], it represents an interesting scenario (associated with potentially strong barrier effects) where our approximations may break down due to significant negative LD within subpopulations. Thus, generalizing the present analysis to arbitrary patterns of epistasis, including various forms of cryptic epistasis (which can generate strong RI even in the absence of strong ecological differentiation; see [52]), remains an interesting direction for future work.

The second key assumption is that even when drift has appreciable effects on allele frequencies, it has no effect on the dynamics of genotypes with multiple selected alleles, allowing us to approximate the latter via a *deterministic* effective migration rate. This second kind of *separation of timescales* emerges naturally when local adaptation is polygenic: selection per locus must then be at least as strong as drift for local adaptation to be possible, i.e., $s≳\frac{1}{2N}\text{log}\left(\frac{1-\rho }{\rho }\right)$, when loci are unlinked [38]. However, drift must then be much weaker than net selection against introgressing genotypes that carry multiple deleterious alleles, i.e., 1/*N* ∼ *s* ≪ *Ls*. This allows us to use fundamentally different mathematical descriptions (deterministic vs. stochastic) for multi-locus and single-locus evolutionary dynamics. In practice, our approximations are accurate for even modestly polygenic architectures (e.g., see the *L*=10 plots in Figs 1A and 2A and 2B).

The basic approach of approximating the effects of LD via an effective migration rate *m*_*e*_ is particularly useful for divergence based on quantitative traits, since *m*_*e*_ then depends primarily on the RV of migrants (see above). This can be estimated in the field, e.g., when pedigrees are available for ∼10 generations (see e.g., [34], [53]), thus providing (in principle) another estimate of the strength of the genomewide barrier to gene flow. The interpretation of effective migration rates in terms of RVs also suggests that at least for highly polygenic architectures of local adaptation, the gross effects of multi-locus LD on allele frequencies may depend on very few quantities, e.g., the mean fitness of *F*_1_ individuals and their within-family variance, even with an arbitrary effect size distribution [54]. Generalizing approximations based on effective migration rates to diploidy, unequal effect sizes and arbitrary dominance is a promising direction, as it will allow us to understand how hybridisation outcomes are influenced by selective interference between loci under different kinds of selective constraint, e.g., loci that are divergently selected (which may generate hybridisation load) and loci under background selection (which may contribute to heterosis).

## Supporting information

S1 Text Supplementary information. (PDF)

## References

[1] Buckler E. S., Holland J. B., Bradbury P. J., Acharya C. B., et al. 2009. The genetic architecture of maize flowering time. Science 325:714–718. doi: 10.1126/science.1174276 19661422

[2] Lamichhaney S., Barrio A. M., Rafati N., Sundström G., et al. 2012. Population-scale sequencing reveals genetic differentiation due to local adaptation in atlantic herring. Proceedings of the National Academy of Sciences 109:19345–19350. doi: 10.1073/pnas.1216128109 23134729 PMC3511109

[3] Lind B. M., Menon M., Bolte C. E., Faske T. M., and Eckert A. J. 2018. The genomics of local adaptation in trees: are we out of the woods yet? Tree Genetics and Genomes 14. doi: 10.1007/s11295-017-1224-y

[4] Soria-Carrasco V., Gompert Z., Comeault A. A., Farkas T. E., et al. 2014. Stick insect genomes reveal natural selection’s role in parallel speciation. Science 344:738–742. doi: 10.1126/science.1252136 24833390

[5] Barton N. H. 1983. Multilocus clines. Evolution 37:454–471. doi: 10.1111/j.1558-5646.1983.tb05563.x 28563316

[6] Feder J. L., Gejji R., Yeaman S., and Nosil P. 2012. Establishment of new mutations under divergence and genome hitchhiking. Philosophical Transactions of the Royal Society B: Biological Sciences 367:461–474. doi: 10.1098/rstb.2011.0256 22201175 PMC3233718

[7] Kawecki T. J. 2008. Adaptation to marginal habitats. Annual Review of Ecology, Evolution, and Systematics 39:321–342. doi: 10.1146/annurev.ecolsys.38.091206.095622

[8] Barton N. H., and De Cara M. A. R. 2009. The evolution of strong reproductive isolation. Evolution 63:1171–1190. doi: 10.1111/j.1558-5646.2009.00622.x 19154394

[9] Butlin R. K., and Smadja C. M. 2018. Coupling, reinforcement, and speciation. The American Naturalist 191:155–172. doi: 10.1086/695136 29351021

[10] Kulmuni J., Butlin R. K., Lucek K., Savolainen V., and Westram A. M. 2020. Towards the completion of speciation: the evolution of reproductive isolation beyond the first barriers. Philosophical Transactions of the Royal Society B: Biological Sciences 375:20190528. doi: 10.1098/rstb.2019.0528 32654637 PMC7423269

[11] Bengtsson B. O. 1985. The flow of genes through a genetic barrier. Pages 31–42 Cambridge University Press Cambridge; New York.

[12] Nagylaki T. 1976. Clines with variable migration. Genetics 83:867–86. doi: 10.1093/genetics/83.4.867 971810 PMC1213557

[13] Barton N., and Bengtsson B. O. 1986. The barrier to genetic exchange between hybridising populations. Heredity 57:357–376. doi: 10.1038/hdy.1986.135 3804765

[14] Via S. 2012. Divergence hitchhiking and the spread of genomic isolation during ecological speciation-with-gene-flow. Philosophical Transactions of the Royal Society B: Biological Sciences 367:451–460. doi: 10.1098/rstb.2011.0260 22201174 PMC3233719

[15] Li W.-H., and Nei M. 1974. Stable linkage disequilibrium without epistasis in subdivided populations. Theoretical Population Biology 6:173–183. doi: 10.1016/0040-5809(74)90022-7 4445973

[16] Slatkin M. 1975. Gene flow and selection in a two-locus system. Genetics 81:787–802. doi: 10.1093/genetics/81.4.787 1213276 PMC1213435

[17] Bürger R., and Akerman A. 2011. The effects of linkage and gene flow on local adaptation: A two-locus continentisland model. Theoretical Population Biology 80:272–288. doi: 10.1016/j.tpb.2011.07.002 21801739 PMC3257863

[18] Geroldinger L., and Bürger R. 2015. Clines in quantitative traits: The role of migration patterns and selection scenarios. Theoretical Population Biology 99:43–66. doi: 10.1016/j.tpb.2014.10.006 25446959 PMC4302420

[19] Feder J. L., and Nosil P. 2010. The efficacy of divergence hitchhiking in generating genomic islands during ecological speciation. Evolution 64:1729–1747. doi: 10.1111/j.1558-5646.2009.00943.x 20624183

[20] Flaxman S. M., Feder J. L., and Nosil P. 2013. Genetic hitchhiking and the dynamic buildup of genomic divergence during speciation with gene flow. Evolution 67:2577–2591. doi: 10.1111/evo.12055 24033168

[21] Feder J. L., Nosil P., Wacholder A. C., Egan S. P., et al. 2014. Genome-Wide Congealing and Rapid Transitions across the Speciation Continuum during Speciation with Gene Flow. Journal of Heredity 105:810–820. doi: 10.1093/jhered/esu038 25149256

[22] Nosil P., Flaxman S. M., Feder Jeffrey L., and Gompert Z. 2017. Tipping points in the dynamics of speciation. Nat Ecol Evol 1:0001. doi: 10.1038/s41559-016-0001 28812620

[23] Yeaman S., and Otto S. P. 2011. Establishment and maintenance of adaptive genetic divergence under migration, selection, and drift. Evolution 65:2123–2129. doi: 10.1111/j.1558-5646.2011.01277.x 21729066

[24] Hill W. G., and Robertson A. 1966. The effect of linkage on limits to artificial selection. Genetical Research 8:269–294. doi: 10.1017/S0016672300010156 5980116

[25] Le Corre V., and Kremer A. 2012. The genetic differentiation at quantitative trait loci under local adaptation. Molecular Ecology 21:1548–1566. doi: 10.1111/j.1365-294X.2012.05479.x 22332667

[26] Yeaman S. 2015. Local adaptation by alleles of small effect. The American Naturalist 186:S74–S89. doi: 10.1086/682405 26656219

[27] Wright S. 1937. The distribution of gene frequencies in populations. Proceedings of the National Academy of Sciences 23:307–320. doi: 10.1073/pnas.23.6.307 PMC1076930 16577780

[28] Petry D. 1983. The effect on neutral gene flow of selection at a linked locus. Theoretical Population Biology 23:300–313. doi: 10.1016/0040-5809(83)90020-5 6623407

[29] Charlesworth B., Nordborg M., and Charlesworth D. 1997. The effects of local selection, balanced polymorphism and background selection on equilibrium patterns of genetic diversity in subdivided populations. Genetical Research 70:155–174. doi: 10.1017/S0016672397002954 9449192

[30] Yeaman S., and Whitlock M. C. 2011. The genetic architecture of adaptation under migration selection balance. Evolution 65:1897–1911. doi: 10.1111/j.1558-5646.2011.01269.x 21729046

[31] Yeaman S., Aeschbacher S., and Bürger R. 2016. The evolution of genomic islands by increased establishment probability of linked alleles. Molecular Ecology 25:2542–2558. doi: 10.1111/mec.13611 27206531

[32] Sakamoto T., and Innan H. 2019. The Evolutionary Dynamics of a Genetic Barrier to Gene Flow: From the Establishment to the Emergence of a Peak of Divergence. Genetics 212:1383–1398. doi: 10.1534/genetics.119.302311 31171654 PMC6707448

[33] Blanquart F., Gandon S., and Nuismer S. L. 2012. The effects of migration and drift on local adaptation to a heterogeneous environment. Journal of Evolutionary Biology 25:1351–1363. doi: 10.1111/j.1420-9101.2012.02524.x 22568832

[34] Chen N., Juric I., Cosgrove E. J., Bowman R., et al. 2019. Allele frequency dynamics in a pedigreed natural population. Proceedings of the National Academy of Sciences 116:2158–2164. doi: 10.1073/pnas.1813852116 PMC6369762 30598449

[35] Good B. H., Walczak A. M., Neher R. A., and Desai M. M. 2014. Genetic diversity in the interference selection limit. PLOS Genetics 10:1–1. doi: 10.1371/journal.pgen.1004222 24675740 PMC3967937

[36] Roze D. 2021. A simple expression for the strength of selection on recombination generated by interference among mutations. Proceedings of the National Academy of Sciences 118. doi: 10.1073/pnas.2022805118 33941695 PMC8126786

[37] Weissman D. B., and Barton N. H. 2012. Limits to the rate of adaptive substitution in sexual populations. PLOS Genetics 8:1–18. doi: 10.1371/journal.pgen.1002740 PMC3369949 22685419

[38] Szép E., Sachdeva H., and Barton N. H. 2021. Polygenic local adaptation in metapopulations: A stochastic eco-evolutionary model. Evolution 75:1030–1045. doi: 10.1111/evo.14210 33742441 PMC8251656

[39] Kobayashi Y., Hammerstein P., and Telschow A. 2008. The neutral effective migration rate in a mainland-island context. Theoretical Population Biology 74:84–92. doi: 10.1016/j.tpb.2008.05.001 18550138

[40] Fisher R. A. 1930. The Genetical Theory of Natural Selection. Oxford: Clarendon Press.

[41] Robertson A. 1961. Inbreeding in artificial selection programmes. Genetical Research 2:189194. doi: 10.1017/S0016672300000690 18976515

[42] Sachdeva H., and Barton N. H. 2018. Replicability of introgression under linked, polygenic selection. Genetics 210:1411–1427. doi: 10.1534/genetics.118.301429 30274989 PMC6283177

[43] Slatkin M. 1991. Inbreeding coefficients and coalescence times. Genetical Research 58:167–175. doi: 10.1017/S0016672300029827 1765264

[44] Roux C., Fraïsse C., Romiguier J., Anciaux Y., Galtier N., and Bierne N. 2016. Shedding light on the grey zone of speciation along a continuum of genomic divergence. PLOS Biology 14:1–22. doi: 10.1371/journal.pbio.2000234 28027292 PMC5189939

[45] Seehausen O. 2004. Hybridization and adaptive radiation. Trends in ecology and evolution 19:198–207. doi: 10.1016/j.tree.2004.05.011 16701254

[46] Brawand D., Wagner C. E., Yang L. I., Malinsky M., and Palma F. D. 2014. The genomic substrate for adaptive radiation in african cichlid fish. Nature 513:375–381. doi: 10.1038/nature13726 25186727 PMC4353498

[47] Hendry A. P., Nosil P., and Rieseberg L. H. 2007. The speed of ecological speciation. Functional Ecology 21:455–464. doi: 10.1111/j.1365-2435.2006.01240.x 19096732 PMC2605086

[48] Latta R. 1998. Differentiation of allelic frequencies at quantitative trait loci affecting locally adaptive traits. The American Naturalist 151:283–292. doi: 10.1086/286119 18811359

[49] Morjan C. L., and Rieseberg L. H. 2004. How species evolve collectively: implications of gene flow and selection for the spread of advantageous alleles. Molecular Ecology 13:1341–1356. doi: 10.1111/j.1365-294X.2004.02164.x 15140081 PMC2600545

[50] Cruickshank T. E., and Hahn M. W. 2014. Reanalysis suggests that genomic islands of speciation are due to reduced diversity, not reduced gene flow. Molecular Ecology 23:3133–3157. doi: 10.1111/mec.12796 24845075

[51] Halligan D. L., and Keightley P. D. 2009. Spontaneous mutation accumulation studies in evolutionary genetics. Annual Review of Ecology, Evolution, and Systematics 40:151–172. doi: 10.1146/annurev.ecolsys.39.110707.173437

[52] Blanckaert A., Bank C., and Hermisson J. 2020. The limits to parapatric speciation 3: evolution of strong reproductive isolation in presence of gene flow despite limited ecological differentiation. Philosophical Transactions of the Royal Society B: Biological Sciences 375:20190532. doi: 10.1098/rstb.2019.0532 32654650 PMC7423268

[53] Hunter D. C., Pemberton J. M., Pilkington J. G., and Morrissey M. B. 2019. Pedigree-Based Estimation of Reproductive Value. Journal of Heredity 110:433–444. doi: 10.1093/jhered/esz033 31259373

[54] Barton N. H., and Etheridge A. M. 2011. The relation between reproductive value and genetic contribution. Genetics 188:953–973. doi: 10.1534/genetics.111.127555 21624999 PMC3176105

