# Supplementary material for: Reproductive isolation via polygenic local adaptation in sub-divided populations: Effect of linkage disequilibria and drift
Source: PLoS Genet. 2022 Sep 1;18(9):e1010297. doi: 10.1371/journal.pgen.1010297 (PMC9473638; doi:10.1371/journal.pgen.1010297)
Supplement: S1 Text — (PDF) [file pgen.1010297.s001.pdf]

SUPPLEMENTARY INFORMATION

Reproductive isolation via polygenic local adaptation in sub-divided populations: effect of linkage disequilibria and drift

Himani Sachdeva

Department of Mathematics, University of Vienna, Vienna 1090, Austria.

Section 1: Effective migration rates (neglecting drift).

Effective migration rate for deleterious alleles. We can solve eq. 3 of the main text iteratively to obtain P_y, the deterministic frequencies of genotypes that carry $y=1,2,\ldots L$ deleterious alleles, in a population at migration-selection equilibrium. These can be expressed as:

$$P_L=m\frac{g_L}{1-g_L}$$

$$P_{L-y}=\binom{L}{y}\frac{P_L}{1-g_{L-y}}\Bigg[1+\sum_{k=1}^{y-1}\binom{y}{k}\frac{g_{L-k}}{1-g_{L-k}}+\sum_{k=1}^{y-2}\sum_{j=1}^{y-k-1}\binom{y}{k}\binom{y-k}{j}\frac{g_{L-k}}{1-g_{L-k}}\frac{g_{L-k-j}}{1-g_{L-k-j}}+$$

$$\sum_{k=1}^{y-3}\sum_{j=1}^{y-k-2}\sum_{v=1}^{y-k-j-1}\binom{y}{k}\binom{y-k}{j}\binom{y-k-j}{v}\frac{g_{L-k}}{1-g_{L-k}}\frac{g_{L-k-j}}{1-g_{L-k-j}}\frac{g_{L-k-j-v}}{1-g_{L-k-j-v}}+\ldots\Bigg],1\leq y\leq L-1$$

$$\text{where}\quad g_k=\frac{e^{-s\,k}}{2^{k-1}} \tag{1}$$

The above equation expresses the genotype frequency P_{L-y} for $y=1,2,\ldots L-1$ as a finite sum over y terms (i.e., the series within the square brackets contains $1,2,3\ldots$ terms in the expressions for P_{L-1}, P_{L-2}, P_{L-3}, $\ldots$ respectively). It is also useful to represent these as infinite sums by Taylor expanding the various $g_k/(1-g_k)$ in powers of g_k. Note that $g_k=\frac{2\,e^{-ks}}{2^k}$ has a simple interpretation: it is the average number of offspring of an individual with k deleterious alleles, who inherit none of the k alleles; alternatively, it is the average number of offspring who inherit

all k alleles. Then, we have the alternative representation:

$$P_y = m\left[\binom{L}{y} g_L + \sum_{k=0}^{L-y}\binom{L}{k}\binom{L-k}{y} g_L g_{L-k} + \sum_{k=0}^{L-y}\sum_{j=0}^{L-k-y}\binom{L}{k}\binom{L-k}{j}\binom{L-k-j}{y} g_L g_{L-k} g_{L-k-j} + \dots\right] \quad (2)$$

Note that the above representation involves an *infinite* series; the kth term in the series is a k-fold convolution. The average deleterious frequency $p_{det} = \sum_{k=1}^{L}(k/L)P_k$ can be obtained by using either eq. (1) or (2). Here, we use eq. (2), thus expressing p_{det} as an infinite sum:

$$\begin{aligned} p_{det} = m &\left[2c\sum_{y=0}^{L-1}\binom{L-1}{y}c^{L-1} + 4c^2\sum_{k=0}^{L-1}\binom{L-1}{k}c^{L-1}\sum_{j=0}^{L-1-k}\binom{L-k-1}{j}c^{L-k-1} + \right.\\ &\left. + 8c^3\sum_{k=0}^{L-1}\binom{L-1}{k}c^{L-1}\sum_{j=0}^{L-k-1}\binom{L-k-1}{j}c^{L-k-1}\sum_{m=0}^{L-k-j-1}\binom{L-k-j-1}{m}c^{L-k-j-1} + \dots\right]\\ &= m(2c)^L\left[1 + (2c)\left(\frac{1+2c}{2}\right)^{L-1} + (2c)^2\left(\frac{1+c+2c^2}{2}\right)^{L-1} + (2c)^3\left(\frac{1+c+c^2+2c^3}{2}\right)^{L-1} + \dots\right]\\ &= m\,(2c)^L\sum_{k=0}^{\infty}(2c)^k\left(\frac{1+c^k-2c^{k+1}}{2(1-c)}\right)^{L-1} \quad \text{where} \quad c = \frac{e^{-s}}{2}\\ &= m\,\frac{e^{-sL}}{(2-e^{-s})^{L-1}}\sum_{k=0}^{\infty}e^{-s\,k}\left(1 + \frac{e^{-sk}}{2^k} - \frac{e^{-s(k+1)}}{2^k}\right)^{L-1} \end{aligned} \quad (3)$$

For $L=1$, the above expression reduces to: $p_{det}(L=1) = m/(e^s-1) \approx m/s$. Following the main text, we define the effective migration rate $m_e(s,L)$ as the migration rate which would cause the deleterious allele frequency at a single locus under migration-selection equilibrium (with selection coefficient s for the deleterious allele) to be equal to p_{det}, which is the average equilibrium frequency that emerges in the multi-locus model, in which immigrant genotypes carrying L such deleterious alleles are introduced at rate m per generation. Then, we have: $m_e(s,L) = p_{det}(e^s-1) \approx s p_{det}$, where p_{det} is given by eq. (3). This finally yields the expression for $m_e(s,L)$ in eq. 4 in the main text.

In the limit $s \to 0$, $L \to \infty$ with $s(L-1) = \theta$ held constant, we can approximate $m_e(s,L)/m$ as:

$$
\begin{aligned}
\frac{m_e(s,L)}{m} &= (1-e^{-s})\left(\frac{e^{-s}}{2-e^{-s}}\right)^{L-1}\sum_{k=0}^{\infty} e^{-s\,k}\left(1+\frac{e^{-sk}}{2^k}-\frac{e^{-s(k+1)}}{2^k}\right)^{L-1} \\
&\approx (1-e^{-s})\,e^{-2\theta}(1+s\,\theta)\sum_{k=0}^{\infty} e^{-s\,k}\left(1+s\left(1-\frac{s}{2}\right)\frac{e^{-sk}}{2^k}\right)^{L-1} \\
&= (1-e^{-s})\,e^{-2\theta}(1+s\,\theta)\sum_{m=0}^{L-1}\binom{L-1}{m}s^m(1-s/2)^m\sum_{k=0}^{\infty}\frac{e^{-sk\,(m+1)}}{2^{k\,m}} \\
&= (1-e^{-s})\,e^{-2\theta}(1+s\,\theta)\sum_{m=0}^{L-1}\binom{L-1}{m}\frac{s^m(1-s/2)^m}{1-\frac{e^{-s\,(m+1)}}{2^{\,m}}} \\
&\approx (1-e^{-s})\,e^{-2\theta}(1+s\,\theta)\left[\frac{1}{1-e^{-s}}+\sum_{m=1}^{L-1}\frac{\theta^m}{m!(1-\frac{1}{2^{\,m}})}\right] \\
&\approx e^{-2\theta}\left[1+s\left(\theta+\sum_{m=1}^{L-1}\frac{\theta^m}{m!}\frac{1}{1-\frac{1}{2^{\,m}}}\right)+\mathcal{O}(s^2)\right] \\
&\approx e^{-2\theta}\left[1+s\left(\theta+\sum_{k=0}^{\infty}\left(e^{\frac{\theta}{2^k}}-1\right)\right)+\mathcal{O}(s^2)\right] \qquad \text{as } s\to 0,\ L\to\infty \text{ with } \theta=s(L-1) \text{ constant}
\end{aligned}
\tag{4}
$$

This is approximated reasonably well by: $m_e(s,L)/m \approx e^{-2\theta}\left[1+s\left(e^{\theta}+e^{\theta/2}-2+\frac{3}{2}\theta\right)\right]$. Thus, the effective migration rate of selected alleles is reduced relative to the raw migration rate by a factor that converges (in the highly polygenic limit) to the average RV e^{-2sL} of migrants (see also the explanation following eq. 5 of the main text).

Effective migration rate for neutral alleles. Following [1], we define the effective migration rate $m_e^*(s,L)$ associated with a neutral locus to be the rate at which neutral alleles embedded within incoming migrant genomes (which carry L unlinked deleterious alleles of effect s) are transferred via one or more recombination events onto the wildtype background (which carry no deleterious alleles) in a population at migration-selection equilibrium.

To calculate this, we must first calculate the equilibrium frequencies $\{f_y\}$ of genotypes that carry the neutral allele in conjunction with y deleterious alleles (each with selective effect s), where all alleles (neutral and deleterious) are unlinked. As before, we assume that the frequency of any genotype with deleterious alleles is sufficiently low that quadratic terms of the kind $f_y f_{y'}$ can be neglected. Then f_y satisfy the following equations in steady state:

$$f_y=\frac{1}{2}\left[mw_L\binom{L}{y}\left(\frac{1}{2}\right)^L+\sum_{k=y}^{L}f_kw_k\binom{k}{y}\left(\frac{1}{2}\right)^k\right] \quad \text{where} \quad w_i=2e^{-is} \tag{5}$$

Note that these are very similar to eq. 3 in the main text, except that the right hand side now has an additional factor of 1/2, which is the probability that an individual carrying the neutral allele transmits it to an offspring. Equation (5) can be solved as before; it is again useful to express the various f_y as an infinite series in powers of g_k, so that we have:

$$\begin{aligned} f_y=m\Bigg[&\frac{1}{2}\binom{L}{y}g_L+\frac{1}{2^2}\sum_{k=0}^{L-y}\binom{L}{k}\binom{L-k}{y}g_Lg_{L-k}+ \\ &\frac{1}{2^3}\sum_{k=0}^{L-y}\sum_{j=0}^{L-k-y}\binom{L}{k}\binom{L-k}{j}\binom{L-k-j}{y}g_Lg_{L-k}g_{L-k-j}+\dots\Bigg] \qquad \text{where} \quad g_i=\frac{w_i}{2^i} \end{aligned} \tag{6}$$

The above expression allows us to read off the distribution of t_{esc}, the time taken for the focal (neutral) allele to 'escape' the deleterious background, by setting y=0. Thus, we have:

$$f_0=m\sum_{k=1}^{\infty}\mathcal{P}(t_{esc}=k) \quad \text{where } \mathcal{P}(t_{esc}=1)=\frac{1}{2}\binom{L}{y}g_L, \quad \mathcal{P}(t_{esc}=2)=\frac{1}{2^2}\sum_{k=0}^{y}\binom{L}{k}\binom{L-k}{y-k}g_Lg_{L-k}, \quad \dots \tag{7}$$

These can be used to calculate various moments $\mathbb{E}[t_{esc}^n]$ of the time to escape.

We can also use $\{f_y\}$ to obtain the effective migration rate $m_e^*(s,L)$ for neutral alleles using:

$$\begin{aligned} \frac{m_e^*(s,L)}{m}&=\frac{1}{2m}\left[g_Lm+\sum_{k=1}^{L}g_kf_k\right] \\ &=c^L\Bigg[1+\sum_{k=0}^{L-1}\binom{L}{k}c^{L-k}+\sum_{k=0}^{L-1}\sum_{j=0}^{L-1-k}\binom{L}{k}\binom{L-k}{j}c^{L-k}c^{L-j-k}+ \\ &\qquad \sum_{k=0}^{L-1}\sum_{j=0}^{L-1-k}\sum_{m=0}^{L-1-k-j}\binom{L}{k}\binom{L-k}{j}\binom{L-k-j}{m}c^{L-k}c^{L-j-k}c^{L-j-k-m}+\dots\Bigg] \\ &=c^L\Big(1+[(1+c)^L-1]+[(1+c(1+c))^L-(1+c)^L]+[(1+c(1+c(1+c)))^L-(1+c(1+c))^L]+\dots\Big) \\ &=\left(\frac{c}{1-c}\right)^L\sum_{k=0}^{\infty}\Big[(1-c^{k+1})^L-(1-c^k)^L\Big] \quad \text{where} \quad c=\frac{e^{-s}}{2} \\ &=\left(\frac{e^{-s}}{2-e^{-s}}\right)^L \end{aligned} \tag{8}$$

In the highly polygenic limit $s\to 0$, $L\to\infty$ with $sL=\theta_*$ held constant, this simplifies to $m_e^*(s,L)/m \approx e^{-2\theta_*}[1+s\theta_*+\mathcal{O}(s^2)]$.

Section 2: Calculating average coalescence times and expected F_{ST} in the infinite-island model using the structured coalescent.

To calculate coalescence times, it is useful to consider a population with a finite number D of demes, and then take the $D\to\infty$ limit. There are 5 distinct possibilities for randomly sampling 2 lineages from D demes, where a fraction $\rho<1/2$ of demes belong to the rare habitat and a fraction $1-\rho$ to the common habitat. With probability ρ/D, both lineages will be sampled from the *same* deme (arbitrarily labeled i) in the rare habitat; with probability $(1-\rho)/D$, both will be from the *same* deme i in the common habitat; with probability $\rho(1-1/D)$, lineages will be sampled from two *different* demes (arbitrarily labeled i and j) both in the rare habitat; with probability $(1-\rho)(1-1/D)$, lineages will be from two different demes i and j both belonging to the common habitat; with probability $2\rho(1-\rho)$, lineages will be sampled from two different demes i and j, one belonging to the rare and the other to the common habitat. We denote the 5 coalescence times corresponding to these 5 different configurations for 2 lineages by: $T_{i,i;\,r}$, $T_{i,i;\,c}$, $T_{i,j;\,r,r}$, $T_{i,j;\,c,c}$, and $T_{i,j;\,r,c}$.

The expected values of the various F_{ST} measures (introduced in the main text) can be expressed in terms of the expected coalescence times as follows:

$$
\begin{aligned}
&F_{ST}^{(r)}=1-\frac{\mathbb{E}[T_{i,i;\,r}]}{\mathbb{E}[T_{tot}]} \qquad F_{ST}^{(c)}=1-\frac{\mathbb{E}[T_{i,i;\,c}]}{\mathbb{E}[T_{tot}]}\\
&\text{where} \quad \mathbb{E}[T_{tot}]=\frac{\rho}{D}\mathbb{E}[T_{i,i;\,r}]+\frac{1-\rho}{D}\mathbb{E}[T_{i,i;\,c}]+\rho\left(1-\frac{1}{D}\right)\mathbb{E}[T_{i,j;\,r,r}]\\
&\qquad +(1-\rho)\left(1-\frac{1}{D}\right)\mathbb{E}[T_{i,j;\,c,c}]+2\rho(1-\rho)\mathbb{E}[T_{i,j;\,r,c}]
\end{aligned} \tag{9a}
$$

$$
\begin{aligned}
&F_{ST}^{(r,r)}=1-\frac{\mathbb{E}[T_{i,i;\,r}]}{\frac{1}{2}\mathbb{E}[T_{i,i;r}]+\frac{1}{2}\mathbb{E}[T_{i,j;r,r}]} \qquad F_{ST}^{(c,c)}=1-\frac{\mathbb{E}[T_{i,i;\,c}]}{\frac{1}{2}\mathbb{E}[T_{i,i;c}]+\frac{1}{2}\mathbb{E}[T_{i,j;c,c}]}\\
&F_{ST}^{(r,c)}=1-\frac{\frac{1}{2}\mathbb{E}[T_{i,j;\,r,r}]+\frac{1}{2}\mathbb{E}[T_{i,j;\,c,c}]}{\frac{1}{4}\mathbb{E}[T_{i,i;r}]+\frac{1}{2}\mathbb{E}[T_{i,j;r,c}]+\frac{1}{4}\mathbb{E}[T_{i,i;c}]}
\end{aligned} \tag{9b}
$$

In order to calculate the expected coalescence times, we consider how lineages trace back in time through different demes belonging to one or other habitat. Let m_{rc} and m_{rr} denote

the probability per unit time that a lineage in a deme within the rare habitat traces back to the rare and common habitats respectively. We can define analogous backward migration rates m_{cc} and m_{cr} for lineages in demes belonging to the common habitat. We use uppercase letters to denote the corresponding population-size-scaled migration rates $M_{rr}{=}Nm_{rr}$, $M_{rc}{=}Nm_{rc}$, $M_{cc}{=}Nm_{cc}$, $M_{cr}{=}Nm_{cr}$. Note that the rates m_{rr} and m_{cc} (or M_{rr} and M_{cc}), as defined here, include backward migration events where a lineage emigrates from a deme but then immigrates back into the same deme (though this occurs with a small probability $1/D$, which vanishes in the limit $D{\to}\infty$).

Going back one generation into the past, there are three possibilities for a pair of lineages that are, at present, in the same deme within the rare habitat: the two lineages can coalesce with probability $1/N$; one or other of the two lineages can trace back to a different deme within the rare habitat with probability $2m_{rr}\left(1-\frac{1}{D\rho}\right)$ (here, the factor $\left(1-\frac{1}{D\rho}\right)$ accounts for the fact that the migration rate m_{rr} also includes 'migration' events which lead a lineage back to its deme of origin); finally, one or other lineage can trace back to a deme belonging to the common habitat with probability $2m_{rc}$. Note that we neglect events involving simultaneous migration of both lineages in a single timestep as these occur with vanishingly small rates under the usual coalescent scaling. Similarly, by enumerating all possible single-generation events for the other 4 two-lineage configurations, we arrive at the following set of recursions for the expected coalescence times:

$$\mathbb{E}[T_{i,i;\,r}]{=}\frac{1{+}2M_{rc}\mathbb{E}[T_{i,j;\,r,c}]{+}2M_{rr}\left(1{-}\frac{1}{\rho D}\right)\mathbb{E}[T_{i,j;\,r,r}]}{1{+}2M_{rc}{+}2M_{rr}\left(1{-}\frac{1}{\rho D}\right)} \tag{10a}$$

$$\mathbb{E}[T_{i,i;\,c}]{=}\frac{1{+}2M_{cr}\mathbb{E}[T_{i,j;\,c,r}]{+}2M_{cc}\left(1{-}\frac{1}{(1-\rho)D}\right)\mathbb{E}[T_{i,j;\,c,c}]}{1{+}2M_{cr}{+}2M_{cc}\left(1{-}\frac{1}{(1-\rho)D}\right)} \tag{10b}$$

$$\mathbb{E}[T_{i,j;\,r,r}]{=}\frac{1{+}2M_{rr}\frac{1}{\rho D}\mathbb{E}[T_{i,i;\,r}]{+}2M_{rc}\mathbb{E}[T_{i,j;\,r,c}]}{2M_{rr}\frac{1}{\rho D}{+}2M_{rc}} \tag{10c}$$

$$\mathbb{E}[T_{i,j;\,c,c}]{=}\frac{1{+}2M_{cc}\frac{1}{(1-\rho)D}\mathbb{E}[T_{i,i;\,c}]{+}2M_{cr}\mathbb{E}[T_{i,j;\,r,c}]}{2M_{cc}\frac{1}{(1-\rho)D}{+}2M_{cr}} \tag{10d}$$

$$\mathbb{E}[T_{i,j;\,r,c}]=\frac{1+M_{cr}\left(1-\frac{1}{\rho D}\right)\mathbb{E}[T_{i,j;\,r,r}]+M_{rc}\left(1-\frac{1}{(1-\rho)D}\right)\mathbb{E}[T_{i,j;\,c,c}]+M_{cr}\frac{1}{\rho D}\mathbb{E}[T_{i,i;\,r}]+M_{rc}\frac{1}{(1-\rho)D}\mathbb{E}[T_{i,i;\,c}]}{M_{cr}+M_{rc}} \tag{10e}$$

Solving eq. (10) for the expectations of the 5 coalescence times, substituting these expressions into eq. (9), and then taking the limit $D\to\infty$ (which allows us to neglect all terms that are $\mathcal{O}(1/D)$) finally gives the expected values of the various F_{ST} measures (eq. 9 of the main text).

Section 3: Convergence to the small s (large L) limit.

As shown in Section 1, in the highly polygenic limit $s\to 0$, $L\to\infty$ with $\theta=(L-1)s$ or $\theta_*=Ls$ fixed, the (scaled) effective migration rates $m_e(s,L)$ and $m_e^*(s,L)$ converge to $m_{e,\infty}(s,L)=m\,e^{-2\theta}$ and $m_{e,\infty}^*(s,L)=m\,e^{-2\theta_*}$ respectively. Note that in this limit, we also have $\theta\to\theta_*$. However, I still distinguish between the two to highlight the conceptual distinction that the barrier effect at any selected locus is due to the other $L-1$ selected loci, while the barrier effect at a neutral locus is due to L selected loci.

As discussed in Section 1, for fixed θ or θ_*, the lowest order (in s) correction to the asymptotic prediction gives (see also eq. 6 of the main text):

$$\begin{aligned}\frac{m_e(s,L)}{m_{e,\infty}(s,L)} &= 1+s\left(\theta+\sum_{k=0}^{\infty}\left(e^{\frac{\theta}{2^k}}-1\right)\right)+\mathcal{O}(s^2)\\ &\approx 1+s\left(e^{\theta}+e^{\theta/2}+\frac{3}{2}\theta-2\right)+\mathcal{O}(s^2) \qquad \theta=(L-1)s\end{aligned} \tag{11a}$$

$$\frac{m_e^*(s,L)}{m_{e,\infty}^*(s,L)}=1+s\,\theta_*+\mathcal{O}(s^2) \qquad \theta_*=Ls \tag{11b}$$

Fig A illustrates this convergence by plotting $(m_e/m_{e,\infty})-1$ for selected alleles (filled symbols) and $(m_e^{(*)}/m_{e,\infty}^{(*)})-1$ for neutral alleles (empty symbols) vs. L for different values of θ or θ_* (different colors). The small s (or equivalently, large L) approximations in equations (11a) and (11b) are shown using solid and dashed lines. As expected, both effective migration rates converge towards the highly polygenic prediction as L increases (or equivalently, s decreases).

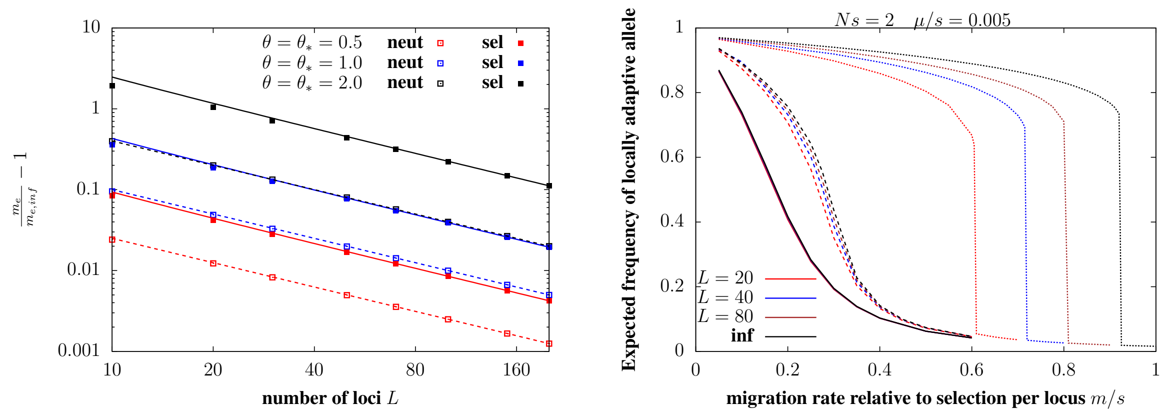

(i) (ii)

Fig A: (i) Relative deviations $m_e/(m_{e,\infty})-1$ (filled squares) and $m_e^*/(m_{e,\infty}^*)-1$ (empty squares) vs. L, the number of selected loci, where m_e and m_e^* are the effective migration rates associated with deleterious and neutral alleles (calculated using eqs. 4 and 5 of the main text), and $m_{e,\infty} = m\, e^{-2\theta}$ and $m_{e,\infty}^* = m\, e^{-2\theta_*}$ the corresponding rates in the highly polygenic limit, obtained by taking $s \to 0$, $L \to \infty$, with $\theta = s(L-1)$ and $\theta_* = sL$ held constant. The different colors correspond to different values of θ or θ_*. Solid and dashed lines show approximate expressions for these deviations (equations (11a) and (11b)), which are correct to first order in s. (ii) The expected frequency of the locally adaptive allele on the island vs. m/s, under mainland-island migration. Solid, dashed and dotted lines correspond to θ=0.5, 1 and 2 respectively; the different colors correspond to different values of L; other parameters are Ns=2 and μ/s=0.001. All finite L predictions in (ii) are obtained by numerically solving eq. 7 in conjunction with eq. 4 of the main text; the predictions for highly polygenic architectures (in black) are obtained by numerically solving eq. 7 while using $m_{e,\infty} = e^{-2\theta}$ for the effective migration rate.

Moreover, this convergence is captured quite accurately by the approximations above.

Note that for a given Ls, the barrier effect is weaker, i.e, m_e higher, when the total selective disadvantage is due to a small number of loci of relatively strong effect. Conversely, the barrier effect is strongest when the total selective disadvantage is due to very many infinitesimal loci.

One can also ask: to what extent is local adaptation sensitive to L (or s) for a given total selective disadvantage Ls, i.e., (when) does the detailed genetic architecture of local adaptation matter? Fig A(ii) shows theoretical predictions for the frequency of the locally adaptive allele on the island vs. m/s for various numbers L of divergently selected loci (various colors), for fixed $\theta = (L-1)s$, and fixed Ns. The finite L predictions are obtained by solving eq. 7 of the main text, using the expression for m_e in eq. 4, while the $L \to \infty$ predictions use the asymptotic expression $m_e(s, L) = m\, e^{-2\theta}$ instead of eq. 4. Fig A(ii) shows these plots for θ=0.5 (solid lines), θ=1.0 (dashed lines) and θ=2 (dotted lines). We see that allele frequencies are more sensitive to the actual genetic architecture (i.e., to L and s individually) when θ is large (dotted lines). Conversely, for smaller θ, local adaptation depends on s only via the composite parameters Ls, Ns and m/s.

In fig. A(ii), s is decreased (while also simultaneously decreasing m and μ and increasing

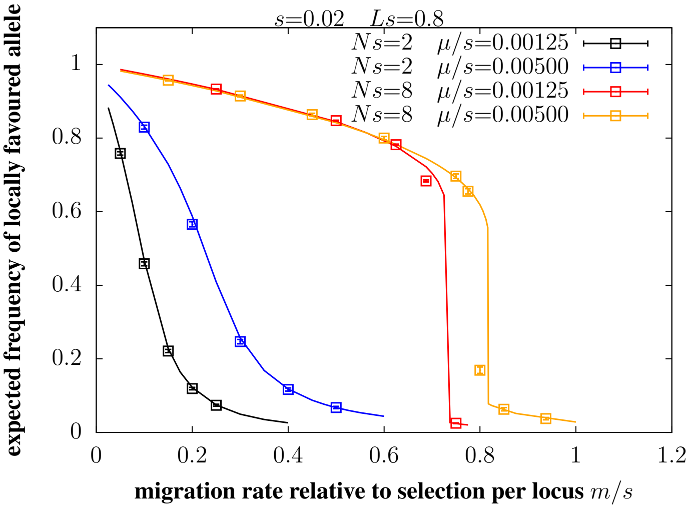

Fig B: Expected frequency of the locally adaptive allele on the island vs. m/s, migration rate relative to selection per locus for two different population sizes (Ns=2 and Ns=8) and two different mutation rates (μ/s=0.00125 and 0.005) for each. Other parameters are: s=0.02, Ls=0.8. Results of individual-based simulations (symbols) are in good agreement with theoretical predictions (lines) obtained from eqs. 7 and 4 of the main text for both mutation rates.

L and N) in order to keep the scaled parameters Ns, m/s, μ/s and the total selective effect Ls constant, since the goal is to illustrate how in the highly polygenic limit, evolutionary outcomes depend on s only via these composite parameters. However, in order to investigate how evolutionary outcomes depend on the genetic architecture of local adaptation for a given total selective disadvantage Ls in a population of a certain size N and subject to a certain rate of migration m, we must compare scenarios where lower s implies lower Ns and higher m/s. Thus, even though more polygenic architectures can, in principle, generate stronger multi-locus barrier effects (see, e.g., fig. A(i)), they are also associated with reduced efficacy of selection and increased swamping at individual loci (via reduced Ns and increased m/s), making local adaptation (in our model) more difficult for more polygenic architectures.

Section 4: Effect of mutation rate on local adaptation in the mainland-island model.

In the main paper, the mutation rate for the mainland-island model was held fixed at μ/s=0.005. Fig B contrasts adaptation thresholds shown in the main paper with those for μ/s=0.00125 (mutation rate lower by a factor of 4), for two different population sizes corresponding to Ns=2 and Ns=8. We see that theoretical predictions that account for both LD and drift (obtained from eq. 7 in conjunction with eq. 4 of the main paper) are quite accurate, also for the lower

mutation rate (lines vs. symbols in fig. B)

As expected, local adaptation is more sensitive to mutation in the smaller (Ns=2) population, with adaptive allele frequencies being significantly lower for the lower mutation rate across all migration levels. Thus, in the smaller population, mutation is instrumental in countering the loss of local adaptation due to drift and gene flow. By contrast, in the larger (Ns=8) population, the adaptive allele frequency is actually marginally higher for the lower mutation rate for $m/s \lesssim 0.5$. Thus, in this regime, mutation has very little effect on polymorphism unless mutation rates are extremely small; e.g., μ/s must be at least as low as $\sim 10^{-6}$ for m/s=0.2, and $\sim 2\times10^{-5}$ for m/s=0.4, to have an appreciable effect on the adaptive allele frequency in the larger population. Sensitivity to mutation rates does, however, increase closer to the threshold for loss of local adaptation, with the threshold m/s falling by about 12% when the mutation rate is reduced from 0.005 to 0.00125. We also find that local adaptation is insensitive to mutation rates over a wider range of m when Ls is larger, as effective migration rates (which govern the extent of maladaptive gene flow) can then be much lower than m.

Section 5: Deterministic analysis of local adaptation in the infinite-island model.

As in the main paper, let p denote the frequency of the allele that is advantageous in the common (and disadvantageous in the rare) habitat. In the deterministic limit, i.e., neglecting drift, we can write down coupled equations for the time evolution of p_r and p_c, the allele frequencies in the rare and common habitats:

$$\begin{aligned} \frac{dp_r}{dt} &= -sp_rq_r + m_e(1-\rho)(p_c-p_r) \\ \frac{dp_c}{dt} &= sp_cq_c + m_e\rho(p_r-p_c) \end{aligned} \tag{12}$$

The assumption here is that allele frequency evolution at any locus is governed by the balance between selection at that locus and migration which is described by an effective migration rate m_e, which captures the 'barrier effect' due to selection against deleterious alleles at the other $L-1$ loci. We will use $\Delta = p_c - p_r$ to denote the allele frequency difference between the two habitats, and express the effective migration rate as $m_e = m\, g[\Delta]$. Here, $g[\Delta]$, which is the gene flow factor, is assumed to depend on the allele frequencies p_c and p_r only via the difference Δ

(see also main paper).

One can employ various approximations for $g[\Delta]$ (see below) and solve eq. (12) numerically to obtain p_c and p_r. The deterministic predictions for m_c/s (the threshold for local adaptation in the rare habitat) in figs. 2c and 2d in the main text are obtained in this way— by using $g[\Delta] \approx e^{-2sL\Delta}[1+s\left(e^{\theta}+e^{\theta/2}+\frac{3}{2}\theta-2\right)]$ (see eq. (11a)), and then numerically solving (12) at equilibrium (i.e., with $dp_c/dt{=}dp_r/dt{=}0$), to determine the value of m_c/s beyond which no non-zero solution for Δ exists. Where multiple equilibria are possible, one can use eq. (12) to determine which equilibrium is reached by numerically solving for $p_r(t)$ and $p_c(t)$ as a function of time t, given initial allele frequencies $p_r(0)$ and $p_c(0)$.

We can also obtain approximate analytical expressions for m_c/s by assuming that the common habitat is always reasonably well-adapted, such that $q_c{=}1{-}p_r$ is small and $\mathcal{O}(q_c^2)$ terms can be neglected. This allows us to approximate: $p_c q_c{\approx}q_c$ and $p_r q_r{\approx}\Delta(1{-}\Delta){+}q_c(1{-}2\Delta)$, which gives the following coupled equations for the time evolution of p_c and Δ.

$$\frac{dp_c}{dt} = s\,q_c - m\,g[\Delta]\,\rho\,\Delta \tag{13a}$$

$$\frac{d\Delta}{dt} = 2sq_c(1-\Delta)+s\Delta(1-\Delta)-m\,g[\Delta]\,\Delta \tag{13b}$$

At migration-selection equilibrium (i.e., assuming the time derivatives in the above equation to be zero), we can use eq. (13a) to express q_c in terms of Δ, and then substitute into (13b) to obtain the following equation for Δ *at equilibrium*:

$$f[\Delta] = \Delta(1-\Delta) - (m/s)\,g[\Delta]\,\Delta(1-2\rho(1-\Delta)) = 0 \tag{14}$$

There is always an equilibrium at $\Delta{=}0$ (corresponding to zero adaptive differentiation between habitats): this is unstable as long as $m/s{<}(1{-}2\rho)$ and is stable otherwise. Note that $m_{c,1}/s{=}(1{-}2\rho)$ is simply the threshold for the maintenance of polymorphism at a single locus (or equivalently, the LE threshold) under deterministic migration-selection equilibrium.

In addition, there may be other equilibria at $\Delta{=}\Delta_*{>}0$ (corresponding to partial local adaptation in the rare habitat), where Δ_* satisfies $1{-}\Delta_*{=}(m/s)[1{-}2\rho(1{-}\Delta_*)]g[\Delta_*]$. While this does not allow us to solve for Δ_* as a function of m/s, we can obtain an explicit expression for the critical migration threshold (denoted by $m_{c,2}/s$) beyond which no such equilibrium (with $\Delta_*{>}0$)

exists. As shown below, the threshold $m_{c,2}/s$ may or may not be equal to the LE threshold $m_{c,1}/s$, depending (primarily) on the values of Ls and ρ.

As a prelude to deriving an expression for $m_{c,2}/s$, it is useful to visualize the behaviour of $f[\Delta]$ as a function of Δ (see eq. (14)). For the purpose of this visualisation, the gene flow factor $g[\Delta]$ is approximated by its asymptotic large L form: $g[\Delta]{\approx}e^{-2Ls\,\Delta}$. However, the qualitative behaviours we observe are not sensitive to this assumption. Figs C(i) and C(ii) show $f[\Delta]$ (scaled by s) vs. Δ for $Ls{=}0.5$ and $Ls{=}1$ respectively, for $\rho{=}0.1$, with the different colors in each plot corresponding to different values of m/s. Those points at which the curves intersect the horizontal axis (i.e., at which $f[\Delta]{=}0$) correspond to equilibria. An equilibrium is *stable* if $f[\Delta]$ is a decreasing function of Δ, i.e., if the curve is downward sloping at the equilibrium, and is unstable otherwise.

For small Ls and low values of m/s (red and brown curves in fig. C(i)), there are two equilibria— an unstable equilibrium at $\Delta{=}0$ and a stable equilibrium at $\Delta{=}\Delta_*{>}0$ (marked by the $\times$ symbol on the plots). Increasing m/s causes Δ_* to decrease; above a critical migration threshold $m_{c,1}/s{=}1/(1{-}2\rho)$, the equilibrium at $\Delta{=}0$ becomes stable while the alternative equilibrium Δ_* becomes unstable and also negative (and thus is no longer biologically meaningful). The transition threshold $m_{c,1}/s{=}1/(1{-}2\rho)$ (blue curve in fig. C(i)), at which divergence between habitats collapses, corresponds to a so-called transcritical bifurcation.

For large Ls (fig. C(ii)), we observe qualitatively different transitions of the equilibria with changing m/s. At low levels of migration, i.e., for $m/s < m_{c,1}/s = 1/(1{-}2\rho)$ (red curve), there is (as before) an unstable equilibrium at $\Delta{=}0$ and a stable equilibrium at $\Delta{=}\Delta_*{>}0$. At intermediate migration levels (brown curve), i.e., for $m_{c,1}/s < m/s < m_{c,2}/s$, the $\Delta{=}0$ equilibrium becomes stable; however, the stable $\Delta_*{>}0$ equilibrium also persists, and there now appears a third (unstable) positive equilibrium separating the two stable equilibria. Increasing m/s now causes the unstable and stable equilibria to converge towards a common value Δ_c. At a critical migration threshold $m_{c,2}/s$ (blue curve), the two equilibria collide (this corresponds to a so-called saddle-node bifurcation), and we have $\Delta_*{=}\Delta_c$ (marked by a circle in fig. C(ii)). Above this threshold, the two non-zero (stable and unstable) equilibria vanish, and there exists only a single stable equilibrium at $\Delta{=}0$ (black curve). Note that $m_{c,2}/s$ is greater than the threshold $m_{c,1}/s$ at which the $\Delta{=}0$ equilibrium becomes stable.

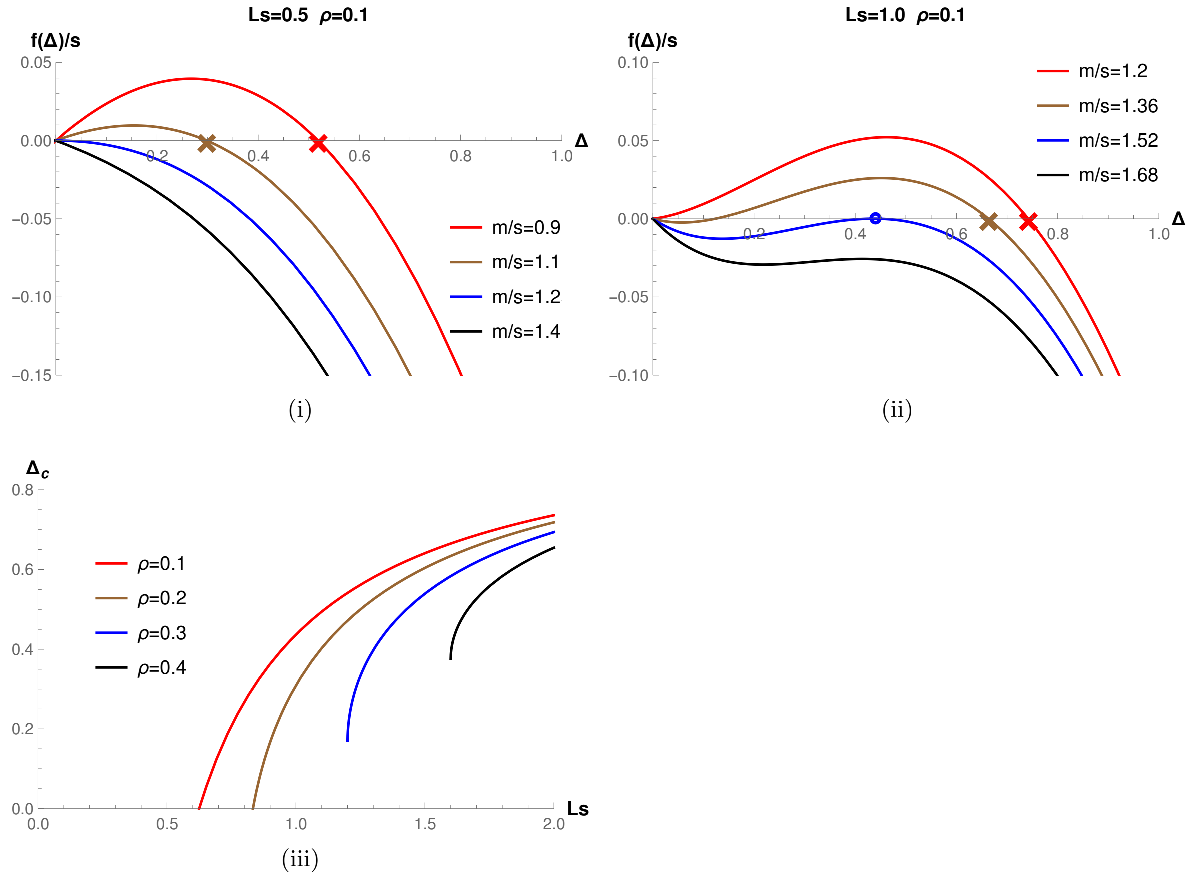

Fig C: Deterministic analysis of local adaptation in the infinite-island model in the highly polygenic limit. (i)-(ii) $f[\Delta]$ (scaled by s) vs. Δ for various values of m/s for ρ=0.1 and (i) Ls=0.5 (ii) Ls=1. Here, Δ=p_c−p_r is the allele frequency difference between the two habitats, and $f[\Delta]$, defined in eq. (13b), determines equilibria. Equilibria are those values of Δ at which $f[\Delta]$=0; stable equilibria are those for which the curve $f[\Delta]$ vs. Δ is downward sloping, i.e., at which $df[\Delta]/d\Delta$ is negative. For low Ls (fig. i), there is a critical migration level $m_{c,1}/s$=$1/(1-2\rho)$ (blue curve), above which the Δ=0 equilibrium becomes stable, while the Δ>0 equilibrium (marked by $\times$ symbols) vanishes. For high Ls (fig. ii), there are two thresholds: the Δ=0 equilibrium becomes stable for m/s>$1/(1-2\rho)$ but the Δ>0 equilibrium also persists, along with a third unstable equilibrium. At a second migration threshold $m_{c,2}/s$ (blue curve), the stable and unstable equilibrium collide: the corresponding value of Δ at which this occurs is Δ_c (marked by a circle in fig. ii), which is the lowest possible divergence level that is stable (for m>$m_{c,1}$). Above the migration threshold $m_{c,2}$, only a single stable equilibrium at Δ=0 exists. (iii) The critical adaptive divergence, Δ_c (obtained from eq. (16a)), vs. Ls for various ρ. There is a threshold $(Ls)_*$ (which increases with increasing ρ) such that Δ_c is non-zero only for Ls>$(Ls)_*$.

We can determine Δ_c by noting that it is that value of Δ at which both $f[\Delta]$ and its first derivative with respect to Δ become zero (note the shape of the blue curve at this equilibrium). Using eq. (14), the two conditions— $f[\Delta_c]$=0 and $f'[\Delta_c]$=0— imply respectively:

$$1-\Delta_c-(m_{c,2}/s)\,[1-2\rho(1-\Delta_c)]\,g[\Delta_c]=0 \tag{15a}$$

$$1-2\Delta_c-(m_{c,2}/s)\,[1-2\rho(1-2\Delta_c)]\,g[\Delta_c]-(m_{c,2}/s)\,\Delta_c\,[1-2\rho(1-\Delta_c)]\,g'[\Delta_c]=0 \tag{15b}$$

Consider first the highly polygenic limit ($s\to 0$, $L\to\infty$ and Ls constant), in which the gene flow factor can be approximated as $g[\Delta]\approx e^{-2Ls\Delta}$, so that we have: $g'[\Delta]=-2Ls\,g[\Delta]$. Substituting into eq. (15) yields a simple quadratic equation in Δ_c, which involves only on Ls and ρ; solving this for Δ_c and then substituting into eq. (15a) gives $m_{c,2}/s$. Thus, in this limit, we have the following expressions for the critical migration threshold and the critical divergence level:

$$\Delta_c^{(0)}=\frac{Ls(4\rho-1)+\sqrt{Ls(Ls-4\rho)}}{4\rho Ls} \tag{16a}$$

$$\frac{m_{c,2}^{(0)}}{s}=\frac{Ls-2\rho-\sqrt{Ls(Ls-4\rho)}}{4\rho^2}\exp\left[\frac{Ls(4\rho-1)+\sqrt{Ls(Ls-4\rho)}}{2\rho}\right] \tag{16b}$$

Fig C(iii) shows the prediction $\Delta_c^{(0)}$ as a function of Ls for various values of ρ. Note that there exists a non-zero $\Delta_c^{(0)}$ only above a threshold value of Ls, which we denote by $(Ls)_*$. For $\rho<1/4$, the critical divergence level $\Delta_c^{(0)}$ decreases as Ls decreases, approaching 0 as $Ls\to(Ls)_*$. This allows us to solve for $(Ls)_*$ by setting $\Delta_c^{(0)}=0$ in eq. (16a), which gives $(Ls)_*=1/[2(1-2\rho)]$ for $\rho<1/4$. For $\rho>1/4$, the critical divergence level $\Delta_c^{(0)}$ approaches a non-zero value as $Ls\to(Ls)_*$: from eq. (16a), it follows that this bifurcation must occur at $Ls=4\rho$. Thus, for $\rho>1/4$, we have $(Ls)_*=4\rho$, with $\Delta_c^{(0)}\to 1-1/(4\rho)$ as $Ls\to(Ls)_*$. This thus provides the deterministic prediction for the threshold $(Ls)_*$ above which LD can sustain local adaptation migration at rates higher than the single-locus critical migration threshold. This prediction for $(Ls)_*$ is shown using dashed vertical lines in figs. 2C and 2D of the main paper.

We can obtain a better approximation for $m_{c,2}/s$ and Δ_c (which is correct to first order in s) by using the expression in eq. (11a) for the gene flow factor. For generality, let us express this as: $g[\Delta]=e^{-2Ls\Delta}(1+s\,g_1[\Delta])$; in all numerics, we will use $g_1[\Delta]=e^{Ls\Delta}+e^{Ls\Delta/2}-2+$

$(3/2)\Delta$. Substituting this into equation (15) does not yield an exact explicit solution for Δ_c and m_c/s. However, one can obtain an approximate solution using a perturbative expansion in s: this involves expressing $m_{c,2}/s$ and Δ_c as $(m_{c,2}/s)=(m_{c,2}^{(0)}/s)+s\,\epsilon_1$ and $\Delta_c=\Delta_c^{(0)}+s\,\epsilon_2$, and then solving for the perturbation terms ϵ_1 and ϵ_2. This finally yields:

$$\Delta_c=\Delta_c^{(0)}\left(1-s\frac{g_1'[\Delta_c^{(0)}]}{4Ls\Delta_c^{(0)}\sqrt{Ls(Ls-4\rho)}}\right) \tag{17a}$$

$$\frac{m_{c,2}}{s}=\frac{m_{c,2}^{(0)}}{s}\left(1-s\,g_1[\Delta_c^{(0)}]\right) \tag{17b}$$

Figs 2C and 2D in the main paper show both the asymptotic ($L\to\infty$, $s\to 0$) prediction (eq. (16b)) for the critical migration threshold, and the more accurate (correct to first order in s) prediction in eq. (17b).

Section 6: Dependence of critical migration thresholds on the initial state of the population.

The existence of multiple stable equilibria (for $Ls>(Ls)_*$ and $m_{c,1}<m<m_{c,2}$) in the deterministic model above suggests that in this parameter regime, evolutionary outcomes in the stochastic model (i.e., with finite Ns) may also be quite sensitive to the *initial state* of the population, i.e., to allele frequency differences between demes and/or genetic variation within demes. We can gain insight into this by comparing simulations initialized in different ways, keeping all other parameters constant.

For simplicity, all loci in all demes in the rare habitat are initialized with the same allele frequency (denoted by $p_{r,0}$); similarly, all loci in all demes in the common habitat are initialized with allele frequency $p_{c,0}$. Fig D shows the results of individual-based simulations with 4 different initialization conditions (ICs): $p_{r,0}=0$, $p_{c,0}=1$ (IC1; red), $p_{r,0}=0.5$, $p_{c,0}=0.5$ (IC2; blue), $p_{r,0}=0.8$, $p_{c,0}=0.8$ (IC3; brown) and $p_{r,0}=0.9$, $p_{c,0}=0.9$ (IC4, orange). IC1 corresponds to a scenario of secondary contact where both habitats are perfectly adapted, resulting in maximum possible adaptive divergence and maximum possible barrier effects at $t=0$, i.e., at the start of the simulation. The other 3 initialization conditions correspond to scenarios where there is no adaptive divergence and no barrier effect at $t=0$; however, some degree of adaptive divergence may evolve, depending on the level of initial genetic variation at adaptive loci, which is maximum

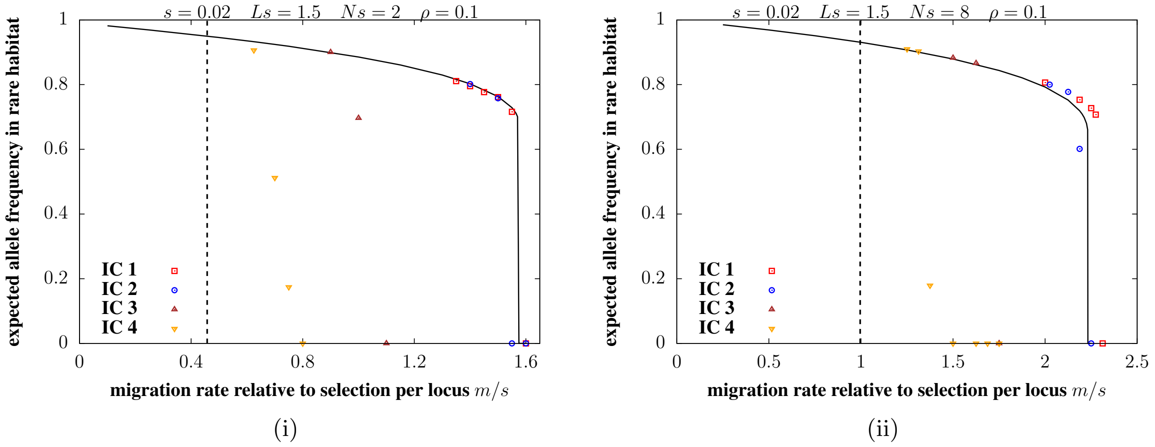

Fig D: Expected frequency of locally adaptive allele in the rare habitat vs. m/s, the migration rate relative to selection per locus for (i) Ns=2 and (ii) Ns=8. Other parameters are: s=0.02, Ls=1.5, ρ=0.1. The four different symbols correspond to results of individual-based simulations initialised with different allele frequencies in the rare and common habitats (see text). The solid black line shows theoretical predictions for the expected allele frequency in the rare habitat- obtained by solving for the polymorphic fixed point of eq. 8 in the main text, using eq. 4. The vertical dashed line represents the LE/single-locus critical migration threshold for a single locus (accounting for drift). Simulation results are obtained by averaging over 5 simulation replicates, each with D=200 demes.

for IC2, and minimum for IC4.

Fig D shows these results for populations of two different size, corresponding to Ns=2 and Ns=8 respectively. We see that long-term evolutionary outcomes are very similar for IC1 (high initial divergence) and IC2 (high initial standing genetic variation, but zero initial divergence). The critical migration threshold for loss of local adaptation in both cases is close to $m_{c,2}$- the theoretically predicted critical migration threshold above which long-term divergence between habitats is not possible, regardless of initial conditions. This threshold can be read off from the solid black curves in figs. D(i) and D(ii)— these show the theoretical predictions for the expected allele frequency, and are obtained using eqs. 8 and 4 of the main paper.

However, critical migration thresholds can be significantly lower when initial frequencies of the locally adaptive allele in the rare habitat are lower (IC3 and IC4), though in these examples, they are still higher than the LE thresholds (vertical dashed lines). Moreover, these thresholds are quite sensitive to initial levels of polymorphism in the population, which determine whether or not populations can evolve a certain minimum level of divergence rapidly enough (despite maladaptive gene flow) for LD-mediated effects to come into play, thus further reinforcing divergence. A more detailed look at simulations for IC3 and IC4 further reveals that close to the threshold for loss of local adaptation, there is considerable heterogeneity across simulation

replicates— some replicates lose all polymorphism, while others evolve a high level of divergence (which is accurately predicted by the theoretical expectations, i.e., solid curve in figs. D(i) and D(ii)). This is in contrast to the deterministic (very high $Ns \rightarrow \infty$) limit, where given certain initial allele frequencies, all replicates are expected to evolve towards the same equilibrium.

References

1. Bengtsson, B. O. 1985. The flow of genes through a genetic barrier. Pages 31-42 Cambridge University Press Cambridge; New York.
